# Dendronized Encapsulation with Hierarchical Rigidification Enabling Robust Solution Room‐Temperature Phosphorescence, Efficient Electroluminescence and Ultralong Afterglow

**DOI:** 10.1002/advs.202511973

**Published:** 2025-08-18

**Authors:** Chensen Li, Zhenchen Lou, Lianrui Hu, Guohua Xie, Song Zhang, Bo Xu, Zheng Zhao, Jacky W. Y. Lam, Ben Zhong Tang

**Affiliations:** ^1^ Key Laboratory for Soft Chemistry and Functional Materials of Ministry of Education School of Chemistry and Chemical Engineering Nanjing University of Science and Technology Nanjing Jiangsu 210094 China; ^2^ Shanghai Key Laboratory of Green Chemistry and Chemical Processes Shanghai Frontiers Science Center of Molecule Intelligent Syntheses School of Chemistry and Molecular Engineering East China Normal University 3663 N. Zhongshan Road Shanghai 200062 China; ^3^ The Institute of Flexible Electronics (Future Technologies) Xiamen University Xiamen Fujian 361005 China; ^4^ State Key Laboratory of Magnetic Resonance and Atomic and Molecular Physics Innovation Academy for Precision Measurement Science and Technology Chinese Academy of Sciences Wuhan Hubei 430071 China; ^5^ School of Science and Engineering Guangdong Basic Research Center of Excellence for Aggregate Science The Chinese University of Hong Kong Shenzhen (CUHK‐Shenzhen) Guangdong 518172 China; ^6^ Department of Chemistry and Hong Kong Branch of Chinese National Engineering Research Center for Tissue Restoration and Reconstruction The Hong Kong University of Science and Technology Kowloon Hong Kong 999077 China

**Keywords:** dendrimers, OLEDs, room‐temperature phosphorescence, solution RTP, ultralong afterglow

## Abstract

Organic room‐temperature phosphorescence (RTP) materials face a fundamental challenge: their environment‐specific phosphorescent behavior fundamentally conflicts with the growing demand for multifunctional materials. To overcome this limitation, a multiscale confinement strategy is developed that integrates intramolecular flexible encapsulation with intermolecular rigid immobilization. Dendronized donor‐acceptor molecules are engineered with alkyl‐chain‐carbazole dendrons to achieve intramolecular encapsulation. This design enables abundant spatial interactions, balancing conformational flexibility (for solution processing) and emission rigidity. Multiscale confinement via molecular aggregation and poly(methyl methacrylate) (PMMA) doping further enhances rigidification. Together, these mechanisms suppress nonradiative transitions across four temporal orders of magnitude (10^−3^–10^0^ s). The resulting material system exhibits unprecedented environment‐adaptive RTP properties: 1) ≈9 ms solution‐phase RTP lifetime under ambient conditions without deoxygenation, representing the longest lifetime among solution‐dissolved RTP systems; 2) 72% photoluminescence quantum yield in doped films and 17.2% external quantum efficiency in organic light‐emitting diodes (OLEDs), making them among the most efficient solution‐processed RTP‐OLEDs; 3) Ultralong afterglow with 1.16 s persistent RTP and 10 s naked‐eye‐detectable emission. Notably, this work represents the first demonstration of a single material simultaneously enabling solution‐phase RTP, high‐efficiency electroluminescence, and long afterglow. This intra/intermolecular engineering overcomes single‐environment limitations, establishing universal design principles for adaptive luminescent materials in various optoelectronic applications.

## Graphical abstract

The organic RTP's rigidity‐flexibility conflict via multiscale confinement is resolved. Dendronized donor‐acceptor with alkyl carbazoles enables intramolecular encapsulation and stability of triplet excitons, achieving 9 ms solution RTP. Combined with aggregation/PMMA doping, this suppresses nonradiative decay, achieving 72% film PLQY/17.2% OLED EQE, and 1.16 s afterglow. This first single‐material platform overcomes environment‐specific limitations.

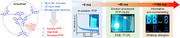

## 1 Introduction

Organic room‐temperature phosphorescence (RTP)^[^ ^1^ ^]^ materials have emerged as transformative candidates for optoelectronic technologies,^[^ ^2^ ^]^ including phosphorescent probes,^[^ ^3^ ^]^ bioimaging,^[^ ^4^ ^]^ solid‐state displays,^[^ ^5^ ^]^ and advanced anti‐counterfeiting systems.^[^ ^6^ ^]^ Their ability to stabilize and harvest triplet excitons for phosphorescence emission through structural rigidification approaches without cryogenic conditions offers unique advantages over conventional fluorescence.^[^ ^7^ ^]^ However, the realization of efficient RTP emission under flexible states or in solution‐phase systems remains a considerable technical hurdle.^[^ ^8^ ^]^ For example, the degrees of freedom of molecular motion in solution are high, and it is easy to consume triplet exciton energy through nonradiative transitions.^[^ ^9^ ^]^ Additionally, triplet excitons are easily quenched by oxygen or moisture in ambient conditions. Traditional strategies such as introducing heavy atoms (e.g., Br, I) can enhance spin‐orbit coupling (SOC) and intersystem crossing (ISC);^[^ ^10^ ^]^ however, these approaches would aggravate nonradiative decay, leading to a shortened afterglow lifetime. Currently, most of the lifetimes of solution‐phase RTP are lower than 2 ms,^[^ ^11^, ^12^, ^13^, ^14^ ^]^ which is not favorable for phosphorescent probes and bioimaging.

In addition, conventional rigidification strategies such as the crystallization,^[^ ^15^ ^]^ supramolecular self‐assembly,^[^ ^16^ ^]^ or doping a rigid matrix^[^ ^17^ ^]^ can physically isolate quenching factors, suppress molecular motion, and reduce nonradiative transitions, achieving high quantum yield and long lifetime.^[^ ^18^ ^]^ Nevertheless, these existing triplet stabilization strategies face an incompatible trade‐off—rigid matrices achieve efficient RTP but compromise film‐forming versatility, while non‐rigid amorphous systems prioritize processability at the expense of phosphorescence efficiency. This rigidity‐flexibility contradiction severely limits their adaptability in multifunctional applications. Current strategies focus on single‐scale modifications, such as internal rigid molecular structures^[^ ^19^ ^]^ or external rigid environment.^[^ ^20^ ^]^ While these approaches partially enhance emission lifetimes, they universally suffer from oxygen sensitivity, low quantum yields, or incompatible electroluminescence properties. Besides, applications of optoelectronic devices such as organic light‐emitting diodes (OLEDs) require materials with both high charge mobility and effective exciton radiative transitions.^[^ ^21^ ^]^ However, rigid RTP molecular structures are beneficial for vacuum‐deposited devices but detrimental to the preparation of solution‐processed RTP‐OLEDs. In contrast, flexible molecular structures are less effective at suppressing nonradiative transitions, leading to reduced solution‐processed device efficiency. These factors result in solution‐processed RTP devices exhibiting significantly lower efficiencies than their vacuum‐deposited counterparts (EQE <10%^[^ ^22^ ^]^ vs >30%^[^ ^23^ ^]^). Although our recent work using conjugated dendrimers achieved a high EQE of 25.1% in solution‐processed OLEDs,^[^ ^24^ ^]^ the solution‐state RTP lifetime remains short (<2 ms),^[^ ^11^, ^12^, ^13^, ^14^ ^]^ while the solid‐state afterglow fails to reach ultralong duration (<40 ms). Significantly, no existing system simultaneously fulfills three major limitations: i) ambient‐stable RTP in solution, ii) high‐efficiency electroluminescence in devices, and iii) naked‐eye‐visible afterglow. The root cause lies in the inability to suppress nonradiative energy losses across multiple time scales (10^−3^–10^0^ s). The lack of a unified design principle to suppress these losses while maintaining structural adaptability represents a critical bottleneck in universal RTP material development.

**Figure 1:**
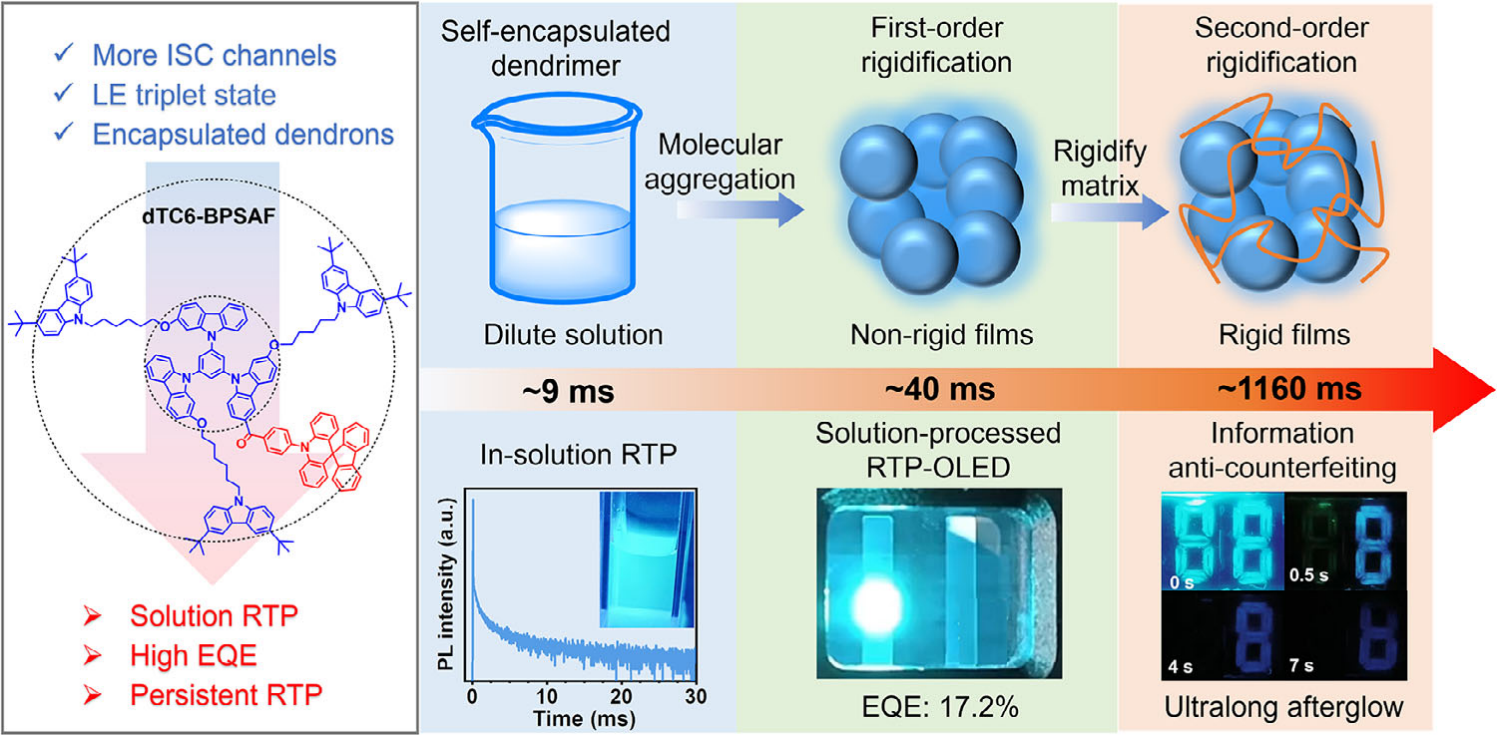
The molecular structure of dTC6‐BPSAF and the RTP properties after being dissolved in solution, host‐doped nonrigid films, and polymer‐doped rigid films.

Herein, we propose a multiscale dynamic rigidity strategy that is beyond conventional single‐scale optimization (**Figure** **1**). By synergistically engineering internal molecular encapsulation and external hierarchical rigidification, we demonstrate a novel single‐material system achieving environment‐adaptive RTP performance. By engineering dendronized thermally activated delayed fluorescence (TADF)^[^ ^25^, ^26^ ^]^ molecules with alkyl chain carbazoles, we achieve intramolecular encapsulation, forming a core‐shell structure. The dendrons not only introduce more triplet splitting and increase ISC channels but also regulate the shift of the lowest triplet states (T_1_) from charge‐transfer (CT) states to localized excited (LE) states. Such structural modifications can significantly enhance ISC efficiency and thereby promote the generation of triplet excitons.^[^ ^24^ ^]^ The long alkyl chain carbazoles enclose the luminescent center within a sterically confined skeleton, reducing the motion of RTP cores and suppressing triplet exciton annihilation between neighboring RTP centers and quenchers (e.g., moisture and oxygen). This structural confinement avoids energy loss and effectively suppresses nonradiative transitions,^[^ ^27^, ^28^ ^]^ thereby extending the triplet lifetime, improving phosphorescence efficiency, and enabling stable RTP in solution. This intramolecular engineering synergizes with intermolecular immobilization through aggregation‐induced rigidification and PMMA matrix doping, collectively suppressing nonradiative pathways across four temporal orders (10^−3^–10^0^ s). The resultant material system breaks multiple performance barriers: 1) ambient solution RTP: ≈9 ms lifetime under air without deoxygenation—surpassing previous oxygen‐sensitive systems by 2 orders of magnitude. 2) Efficient electroluminescence: 72% photoluminescence quantum yield (PLQY) in doped films and 17.2% external quantum efficiency (EQE) in OLEDs, making them among the most efficient solution‐processed RTP‐OLEDs. 3) Persistent afterglow: 1.16 s persistent RTP with 10 s naked‐eye visibility, enabling multilevel temporal encryption. This work validates multiscale dynamic rigidity as a universal design rule for adaptive RTP materials, resolving the long‐standing rigidity‐flexibility conflict. Moreover, it demonstrates unprecedented multifunctionality in a single‐component system—a critical breakthrough toward practical applications. Our strategy opens avenues for RTP materials that dynamically regulate their photophysical behavior across liquid, film, and rigid states, with significant implications for flexible optoelectronics, phosphorescence bioimaging, and anti‐counterfeiting technologies.

## 2 Results and Discussion

### 2.1 Molecular Synthesis and Characterizations

Our search for highly efficient capsule RTP dendrimers commenced with the selection of benzophenone (BP) as the donor and spiro[acridine‐9,9’‐fluorene] (SAF) as the acceptor.^[^ ^29^ ^]^ To effectively encapsulate BPSAF, we introduced 1,3,5‐tris(2‐((6‐(3,6‐di‐*tert*‐butyl‐9*H*‐carbazol‐9‐yl)hexyl)oxy)‐9*H*‐carbazol‐9‐yl)benzene (TC6) as nonconjugated dendrons. The synthetic routes and fundamental characterizations can be found in Scheme S1 and Figures S23–S30 (Supporting Information). dTC6‐BPSAF exhibits excellent thermal stability with high decomposition temperature of 459 °C at 5 wt.% initial weight loss and glass‐transition temperature of 265 °C (Figure S1, Supporting Information). The cyclic voltammetry experiment reveals they have reversible oxidation and reduction processes (Figure S2, Supporting Information). The energy levels of HOMO and LUMO are measured as −5.52 and −2.09 eV, respectively, for dTC6‐BPSAF, from the onsets of oxidation and reduction waves.

### 2.2 Theoretical Calculations

**Figure 2:**
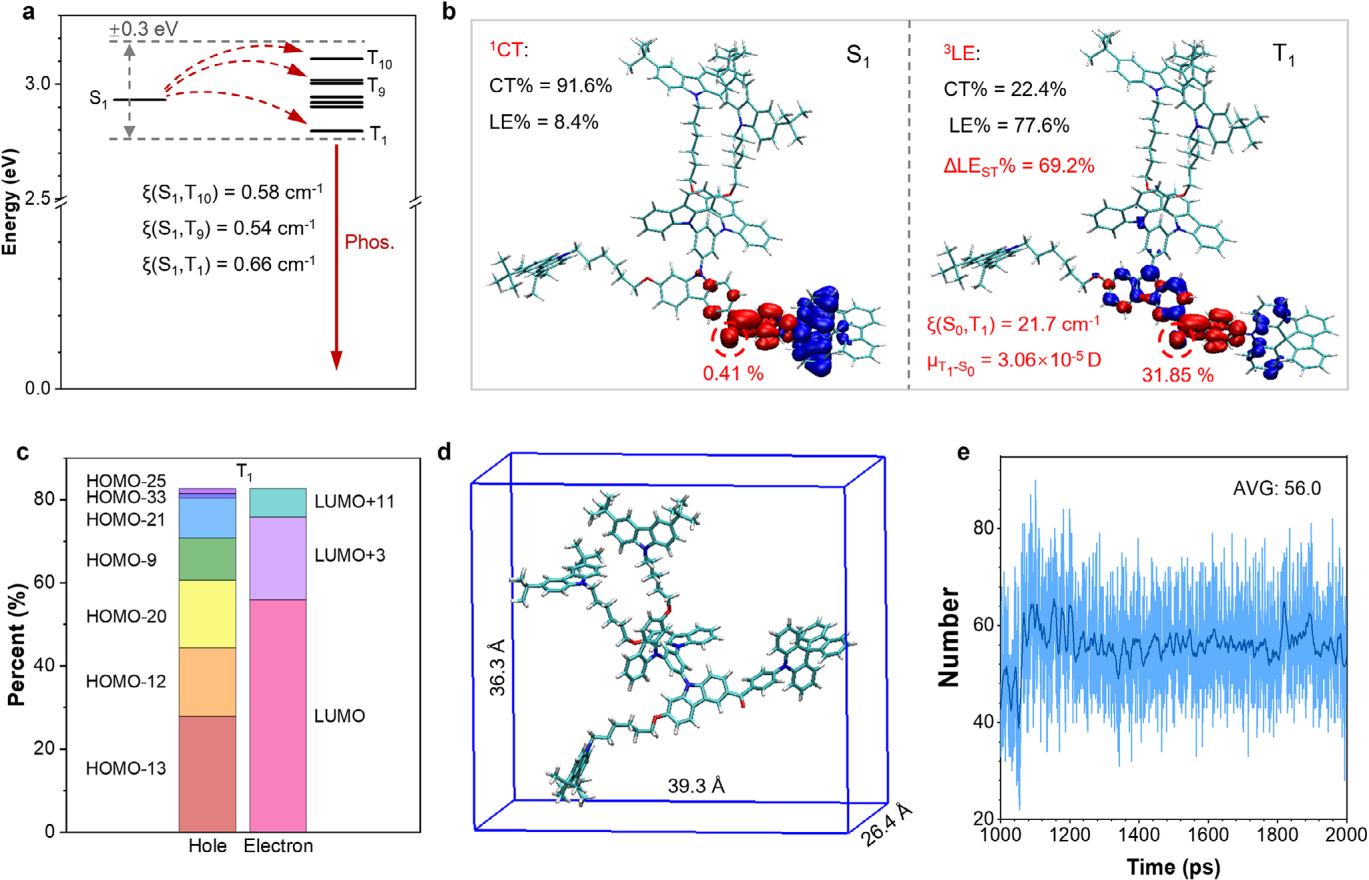
Quantum chemistry and molecular dynamics calculation results of dTC6‐BPSAF. a) The energy levels, ISC processes, and SOC constants of excited states. b) the hole and electron distribution, and the CT and LE proportions of S_1_ and T_1,_ and triplet radiative transition parameters. c) The contributions of HOMOs and LUMOs to the hole and electron in the T_1_ state. d) The length, width, and height of the molecule. e) The number of TSSCs (through‐space short contact with atomic distance *d* < 3.5 Å) from the molecular dynamic simulation.

Two crucial photophysical processes of RTP emission were investigated: 1) the enhancement of ISC spin‐flipping processes from the lowest singlet (S_1_) to the nth triplet state (T*_n_*) (*k*_ISC_), and 2) the facilitation of the phosphorescent decay rate from the lowest triplet (T_1_) to the S_0_ (*k*_P_). The ISC process was evaluated based on Equation (1).^[^ ^24^ ^]^

(1) $$k_{\mathrm{ISC}}\propto \sum_{n}{\left|<S_{1}\left|H_{\mathrm{SOC}}\right|T_{1}>\right|}^{2}\exp\left(-{E^{2}}_{S_{1}T_{n}}\right)$$

where smaller Δ*E*_ST_, coupled with an increased number of ISC channels and larger spin‐orbit coupling operator (${\hat{H}}_{SOC}$) can enhance the ISC rate. First, the dendronization strategy was found to preserve a small overlap between the highest occupied molecular orbitals (HOMOs) and the lowest unoccupied molecular orbitals (LUMOs), located on the SAF donor and BP acceptor moieties, respectively (Figure S3, Supporting Information), resulting in a small Δ*E*_ST_ (0.14 eV), which reduces the energy barrier for the ISC process. Second, the introduction of dendrons reduces the triplet‐triplet energy gaps (Δ*E*_TT_) and increases the density of triplet states, which promotes ISC channels from S_1_ to T_n_. Specifically, the dendrimer dTC6‐BPSAF leads to three valid ISC channels (S_1_→T_1_, S_1_→T_9_, and S_1_→T_10_) (**Figure** **2**a) with both small Δ*E*_ST_ (≤ 0.30 eV) and high SOC values (≥ 0.3 cm^−1^),^[^ ^30^ ^]^ which significantly enhances the ISC efficiency.

In addition to the ISC process, the triplet radiative characteristics (such as SOC (S_1_, T_0_) and $\mu_{T_{1}\to S_{0}}$) are of great significance for evaluating RTP emissions. Specifically, SOC (S_1_, T_0_) can be qualitatively estimated by evaluating the changes in the hole or electron properties of the excited states. In Figure 2b, the majority of hole distribution on BP acceptor, causes the nature of T_1_ to shift from CT states of S_1_ shift to LE states, thereby increasing the variation in the CT transition due to increasing the variation of CT transition between S_1_ and T_1_ (ΔCT_ST_%) and the hole‐electron overlap of 69.2%.^[^ ^31^ ^]^ Meanwhile, the hole‐electron overlap in carbonyl oxygen (overlap(O)) also displays an obvious increase from 0.41% in S_1_ to 31.85% in T_1_. Therefore, this significant increase in the SOC (S_1_, T_0_) constant of 21.7 cm^−1^ enhances the rate of phosphorescence radiative transition (*k*_P_). The *k*_P_ is also determined by the transition dipole moment between the T_1_ and S_0_ states ($\mu_{T_{1}\to S_{0}}$). High triplet densities enhance spin‐allowed T*_n_* →T_1_ transition intensity, enabling a large $\mu_{T_{1}\to S_{0}}$ of 3.06×10^−5^ D. Therefore, the high values of SOC (S_1_, T_0_) and $\mu_{T_{1}\to S_{0}}$ are favorable to efficient RTP emissions.

To explore the origin of the enhanced hole distribution in the T_1_ of the acceptor in dTC6‐BPSAF, the contributions of HOMOs to the hole and LUMOs to the electron in the T_1_ state were analyzed (Figure 2c; Figures S4 and S5, Supporting Information). HOMO‐13 contributes only 27.75% to the hole of T_1_ state, with HOMO‐12, HOMO‐20, and HOMO‐9 accounting for 16.52%, 16.28%, and 10.18%, respectively. Additionally, other orbitals such as HOMO‐21, HOMO‐33, and HOMO‐25 account for more than 1%. In contrast, the contribution rate of LUMO to electrons is as high as 55.76%, and LUMO+3's contribution rate to electrons is 20.00%. These results indicate that dendronization can generate more molecular orbitals for hole distributions than for electron distributions, which is beneficial to enhance ΔCT_ST_% and generate a ^3^LE state, improving the ISC process and phosphorescence radiative transition. Hence, the dendronization strategy holds the potential to convert TADF into efficient RTP by enhancing both the *k*_ISC_ and *k*_p_ achieved through the modulation of triplet energy levels and characteristics.

In addition to generating triplet excitons, stable triplet excitons are also crucial for efficient RTP emission. A core‐shell dendronized structure with alkyl chain carbazoles can enclose the luminescent center within a confined skeleton, reduce the motion of the luminescent unit, and suppress triplet exciton annihilation between external quenchers or closed luminescent centers. The molecular size of 39.3 × 26.4 × 36.3 Å of dTC6‐BPSAF (Figure 2d) is much larger than that of the corresponding small molecules^[^ ^29^ ^]^ and conjugated dendrimers,^[^ ^24^ ^]^ indicating the potential of abundant intramolecular interactions due to the encapsulated alkyl chain carbazole dendrons. Moreover, the intramolecular interactions (atomic distance *d* < 3.5 Å)^[^ ^32^ ^]^ of single molecules can be estimated by calculating the number of through‐space short contacts (TSSCs) of dTC‐BPSAF based on molecular dynamics (MD) simulations using the GROMACS 2023.1 package^[^ ^33^ ^]^ (Figure 2e). The average of TSSCs between the D‐A core (red color) and dendrons (blue color) (Figure 1) for dTC6‐BPSAF is 56.0, indicating a significantly increased intramolecular interaction due to the encapsulated alkyl‐chain‐carbazole dendrons. Therefore, the abundant intramolecular interactions of dTC6‐BPSAF effectively encapsulate the luminescent unit with restricting the molecular motion of the D‐A center and stabilizing the triplet excitons for emitting RTP in a single‐molecular state.

### 2.3 Photophysical Properties in Solution

**Figure 3:**
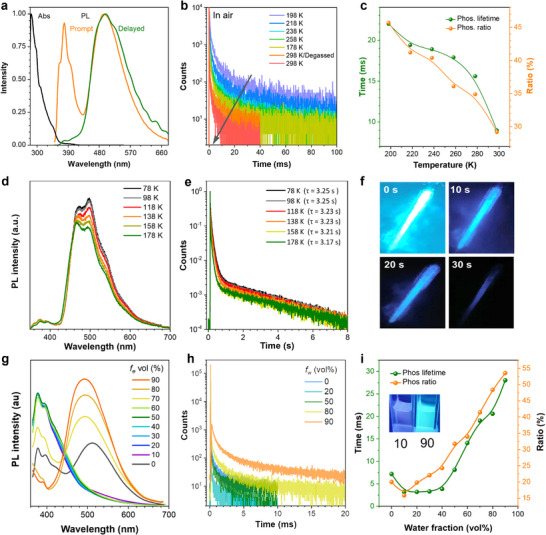
Photoluminescence (PL) behaviors of dTC6‐BPSAF in solutions. a) The absorption (Abs) spectrum and the prompt and delayed PL spectra of dTC‐BPSAF in toluene (10 µm) at room temperature. b) The temperature‐dependent transient phosphorescence decay curves at λ_em_ = 492 nm in toluene. c) The plots of phosphorescence lifetimes and proportions at different temperatures. d) The temperature‐dependent phosphorescence spectra of solidified in toluene, excited at 330 nm. Delay time: 1 ms. e) The temperature‐dependent phosphorescence lifetime curves in solidified toluene with emission recorded at the maximum peak of phosphorescence. f) Photographs in solidified toluene were captured at 77 K before and after the removal of the 365 nm UV light source. g) PL spectra in water/THF mixtures with different water fractions (*f*_w_). h) Transient PL decay spectra in water/THF mixtures with *f*_w_. i) The plot of the lifetimes and proportions of phosphorescence in different ratios of mixtures solutions.

The encapsulated RTP dendrimer dTC6‐BPSAF was successfully synthesized. The UV–vis absorption spectra (**Figure** **3**a) reveal the presence of an intramolecular charge‐transfer (ICT) band spanning ≈370–400 nm, indicating an obvious ICT property of dTC6‐BPSAF. In the photoluminescence (PL) spectra in toluene at 298 K, dTC6‐BPSAF exhibits a sky‐blue emission peak centered at 490 nm and a blue fluorescence band originating from the carbazole units at 379 nm. This indicates that the carbazole dendron units do not participate in ICT in dilute solution due to the steric hindrance from the long alkyl chains (Figure S6, Supporting Information). In the phosphorescence spectra with a delayed time of 0.1 ms in toluene at 298 K, dTC6‐BPSAF exhibits a structureless CT‐type emission at 499 nm, suggesting that the long‐lived emission spectra may originate from triplet states. In the transient PL decay spectra in toluene solution (Figure 3b), dTC6‐BPSAF displays a short lifetime (Figure S7, Supporting Information) and a significantly long‐lived lifetime with τ_P_ of 8.97 ms in air‐equilibrated toluene, representing the longest lifetime among solution‐dissolved RTP systems. The 8.97 ms lifetime arises from 56.0 intramolecular TSSCs, which restrict motion and shield triplet excitons. The long‐lived lifetimes and PL intensities (Figure S8, Supporting Information) decrease monotonically with increasing temperature (Figure 3c), which is contrary to TADF and consistent with phosphorescence. Additionally, phosphorescence lifetimes with τ_P_ values of 9.35 ms are also observed in deoxygenated solutions. These results indicate that the RTP exhibited by dTC6‐BPSAF remains highly stable even in the presence of oxygen when dissolved in solution, indicating excellent oxygen resistance and RTP performance of dTC6‐BPSAF in solution. The air‐stable solution‐phase RTP can be attributed to the abundant intramolecular interactions between the encapsulated dendrons and the RTP emission core (Figure 2e), which benefit the protection of triplet excitons from external quenchers. The peak widths of ^1^H NMR spectra of dTC6‐BPSAF display minor changes in CD_2_Cl_2_ from 248 to 298 K (Figure S9, Supporting Information), evidencing the encapsulating effect of the dendrons. The highly branched skeleton of a dendrimer may lead to tight entanglement of alkyl chains, forming a locally rigid environment. Even with an increase in temperature, the rotation or swing of the alkyl chain is still limited by steric hindrance, and the kinetic behavior is insensitive to temperature. Additionally, the alkyl chains and carbazoles of dendrons may synergistically drive molecules to form stable single‐molecule aggregates in solution, with minimal thermal disturbance to their internal structures. Furthermore, the reactive oxygen species (ROS) generation^[^ ^26^ ^]^ (Figure S10, Supporting Information) results display that the absorbance peaks of singlet oxygen (^1^O_2_) indicator exhibit a slight decrease in the presence of dTC6‐BPSAF under light irradiation. These results further suggest that the large molecular volume of encapsulated dendrons isolates oxygen and inhibits triplet exciton quenching, evidencing the successful realization of air‐stable RTP in dTC6‐BPSAF. Moreover, long phosphorescence lifetimes are also observed in various solutions of dTC6‐BPSAF (Figure S11, Supporting Information), indicating the universality of its stable, long‐lived RTP across different solvent environments.

To characterize the cryogenic phosphorescence of dTC6‐BPSAF, we measured its PL spectra in a solidified toluene matrix at low temperatures. The PL spectra exhibited partially resolved LE‐type emission profiles (Figure 3d), which are very similar to phosphorescence spectra of the T_1_ excited state (Figure S12, Supporting Information). As the temperature increased from 78 to 178 K, PL intensities decreased slightly to ≈80%, demonstrating excellent phosphorescence stability enabled by the encapsulated alkyl‐chain‐carbazole dendrons that restrict thermal motion. Meanwhile, transient PL decay measurements revealed ultralong phosphorescence lifetimes under cryogenic conditions: fitting gave a lifetime of 3.25 s at 78 K, which remained stable at 3.17 s even at 178 K (Figure 3e). To visually document the ultralong afterglow phenomenon, time‐resolved photographs of the phosphorescent emission were captured (Figure 3f). These images show that the dTC6‐BPSAF‐toluene solid continued emitting bright phosphorescence for ≈30 s after deactivating the 365 nm UV excitation source. Such exceptional afterglow properties in rigid matrices highlight the dendrimer's potential as a promising material for practical applications requiring persistent luminescence.

To investigate the RTP properties from solution to aggregate states, the PL properties of dendrimers in dilute THF/water solutions were measured. The PL intensity of dTC6‐BPSAF exhibited weak ICT emission at ≈500 nm and strong carbazole emission at ≈380 nm, with water adding to fractions (*f*_w_) of 60%. However, as the *f*_w_ increased to 90%, the PL emissions at ≈500 nm increased significantly by orders of magnitude, indicating a pronounced aggregation‐induced emission^[^ ^34^ ^]^ phenomenon (Figure 3g). Concurrently, the PL lifetimes also exhibited a substantial increase from 3.2 to 28.0 ms with an increasing water solution from 10% to 90% (Figure 3h,i), indicating aggregation‐induced phosphorescence (AIP)^[^ ^35^ ^]^ behaviors. The *k*_nr_ of dTC6‐BPSAF was calculated to be 314 s^−1^ in the 10% water solution and significantly decreased to a *k*_nr_ of 34 s^−1^ in the aggregated state with 90% water (Table S1, Supporting Information), indicating restricted intramolecular motions and the blocking of nonradiative pathways as the degree of aggregation increases. These findings highlight the crucial role of aggregated states in promoting and amplifying the RTP properties of the dendrimer, indicating its potential applications in aggregated states.

### 2.4 Photophysical Properties in Non‐Rigid Films and OLEDs Properties

The dendrimer dTC6‐BPSAF exhibits strong RTP emission in solution because its encapsulated dendrons suppress exciton quenching. Upon the molecular rigidity is increased through spin‐coating to form doped films, the RTP performance is significantly enhanced. The PL spectra of the doped host material 10‐(4‐((4‐(9H‐carbazol‐9‐yl)phenyl)sulfonyl)phenyl)‐9,9‐dimethyl‐9,10‐dihydroacridine (CzAcSF)^[^ ^36^ ^]^ at 10 wt.% in films exhibit a PL emission peak at 479 nm, and a phosphorescence emission peak at 486 nm for dTC6‐BPSAF, respectively (Figure S13, Supporting Information). Furthermore, sufficient spectral overlap between CzAcSF's emission and dTC6‐BPSAF's absorption indicates effective Förster energy transfer from host to emitter, benefiting high‐performance OLEDs (Figure S14, Supporting Information). Temperature‐dependent phosphorescence decay reveals a considerably longer lifetime of 39.4 ms in doped films compared to 8.97 ms in solutions. (Table S2, Supporting Information). Additionally, exciton dynamics demonstrate that dTC6‐BPSAF exhibits significantly higher *k*_ISC_ of 3.39×10^7^ s^−1^, *k*_P_ of 11.4 s^−1,^ and lower *k*_nr_ of 15.5 s^−1^ in doped films than in solution (4.36×10^6^, 2.93, and 109 s^−1^, respectively). In films, dendron encapsulation more effectively restricts molecular motion, reducing nonradiative decay and enhancing ISC efficiency for stronger phosphorescence versus solution. Consequently, dTC6‐BPSAF‐doped films achieved a PLQY of 72%, demonstrating significant potential in fabricating efficient solution‐processed RTP‐OLEDs.

**Figure 4:**
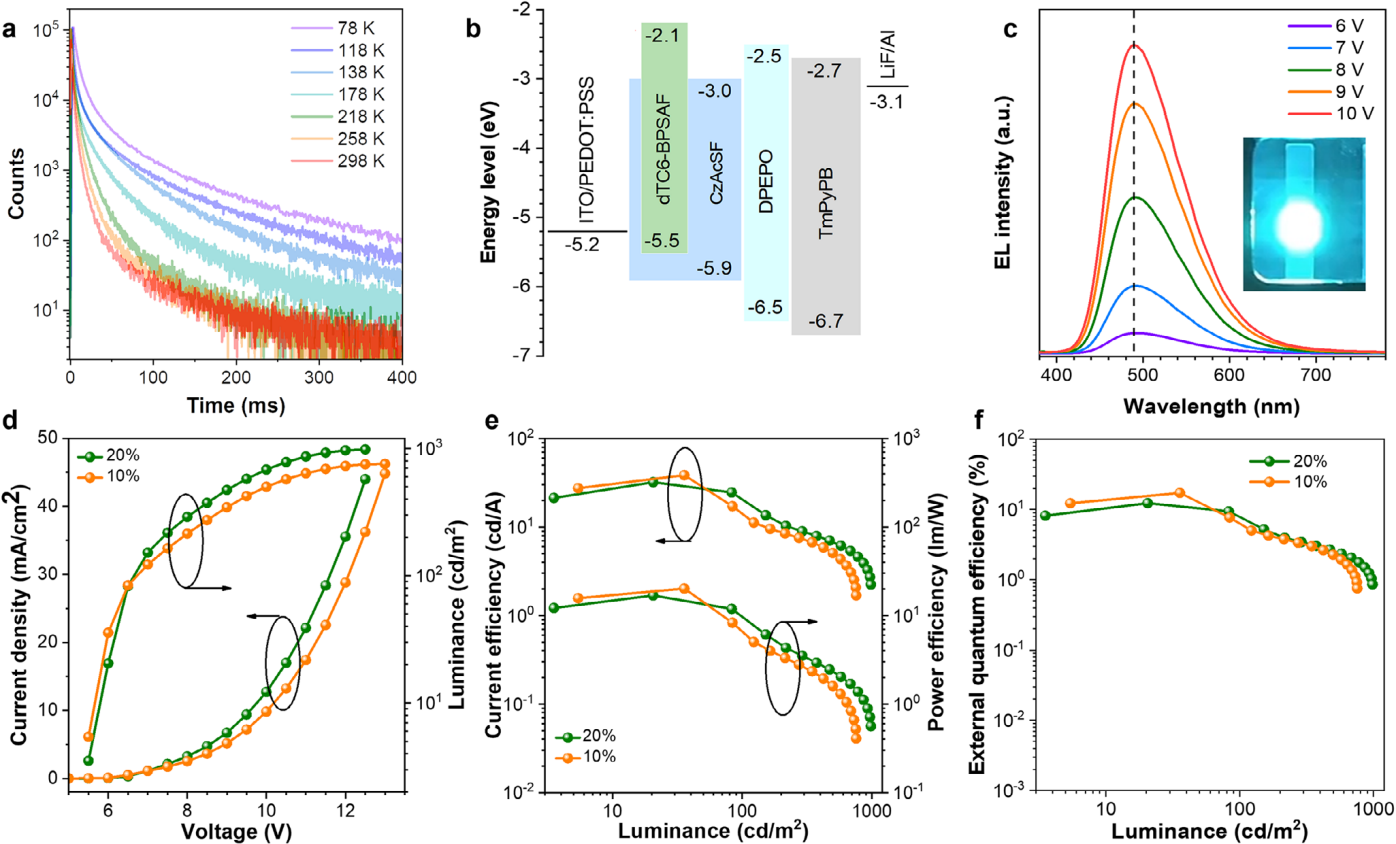
Photophysical behavior and solution‐processed OLEDs performance of dTC6‐BPSAF in doped host films. a) The temperature‐dependent phosphorescence transient decay of dTC6‐BPSAF at λ_em_ = 492 nm in doped CzAcSF films; λ_ex_ = 375 nm. b) The device configuration of the solution‐processed RTP‐OLEDs. c) The EL spectral stability of the RTP‐OLEDs; inset: EL photo of the device measured at 6 V. d) Current density‐voltage‐luminance curves of the devices. e) Current efficiency‐luminance‐power efficiency curves of the devices. f) External quantum efficiency versus luminance curves of the devices.

In this work, the OLED devices were fabricated by solution processing with a configuration of indium tin oxide (ITO)/poly(3,4‐ethylenedioxythiophene): poly(styrenesulfonate) (PEDOT:PSS) (70 nm)/10‐(4‐((4‐(9H‐carbazol‐9‐yl)phenyl)sulfonyl)phenyl)‐9,9‐dimethyl‐9,10‐dihydroacridine (CzAcSF): emitters (10/20 wt.%, 40 nm) /Bis[2‐(diphenylphosphino)phenyl] ether oxide (DPEPO) (10 nm)/1,3,5‐tri(m‐pyrid‐3‐ylphenyl)benzene (TmPyPB) (50 nm)/lithium fluoride (LiF) (1 nm)/aluminum (Al) (100 nm) (**Figure** **4**b). Detailed device data are shown in Table S3 (Supporting Information). The optimized devices with 10% doped exhibited sky‐blue electroluminescence (EL) with a peak wavelength of 492 nm and the CIE color coordinates of (0.18, 0.36). Notably, the EL spectra remained remarkably stable at different voltages, owing to the small Δ*E*_ST_ and near‐complete overlap of fluorescence and phosphorescence spectra (Figure 4c). Similarly, the measured EL lifetime (35.4 ms) approaches the photoluminescence lifetime (37.1 ms), indicating minimal quenching at the charge‐injection interface (Figure S15, Supporting Information). This achievement overcomes the limitations typically associated with conventional RTP devices.^[^ ^37^ ^]^ The current density‐voltage‐luminance curves of the devices are shown in Figure 4d. The devices with 10% doped achieved high maximum current efficiency (CE_max_) of 38.6 cd A^−1^ (Figure 4e), maximum power efficiency (PE_max_) of 20.2 lm W^−1^, and EQE_max_ of 17.2% (Figure 4f), making them among the most efficient solution‐processed RTP‐OLEDs. In addition, the 20% doped devices demonstrated CE_max_ of 32.2 cd A^−1^, PE_max_ of 16.8 lm W^−1^, and EQE_max_ of 12.3%, respectively. Considering that the light out‐coupling efficiency of the conventional OLEDs is 20–30%, the above‐mentioned devices can achieve almost a unit exciton utilization rate. Hence, our design strategy, introducing encapsulated dendrons to generate efficient RTP, exhibits the significant potential to enhance the efficiency of solution‐processed RTP‐OLEDs.

### 2.5 Photoluminescence Properties in Rigid Films

**Figure 5:**
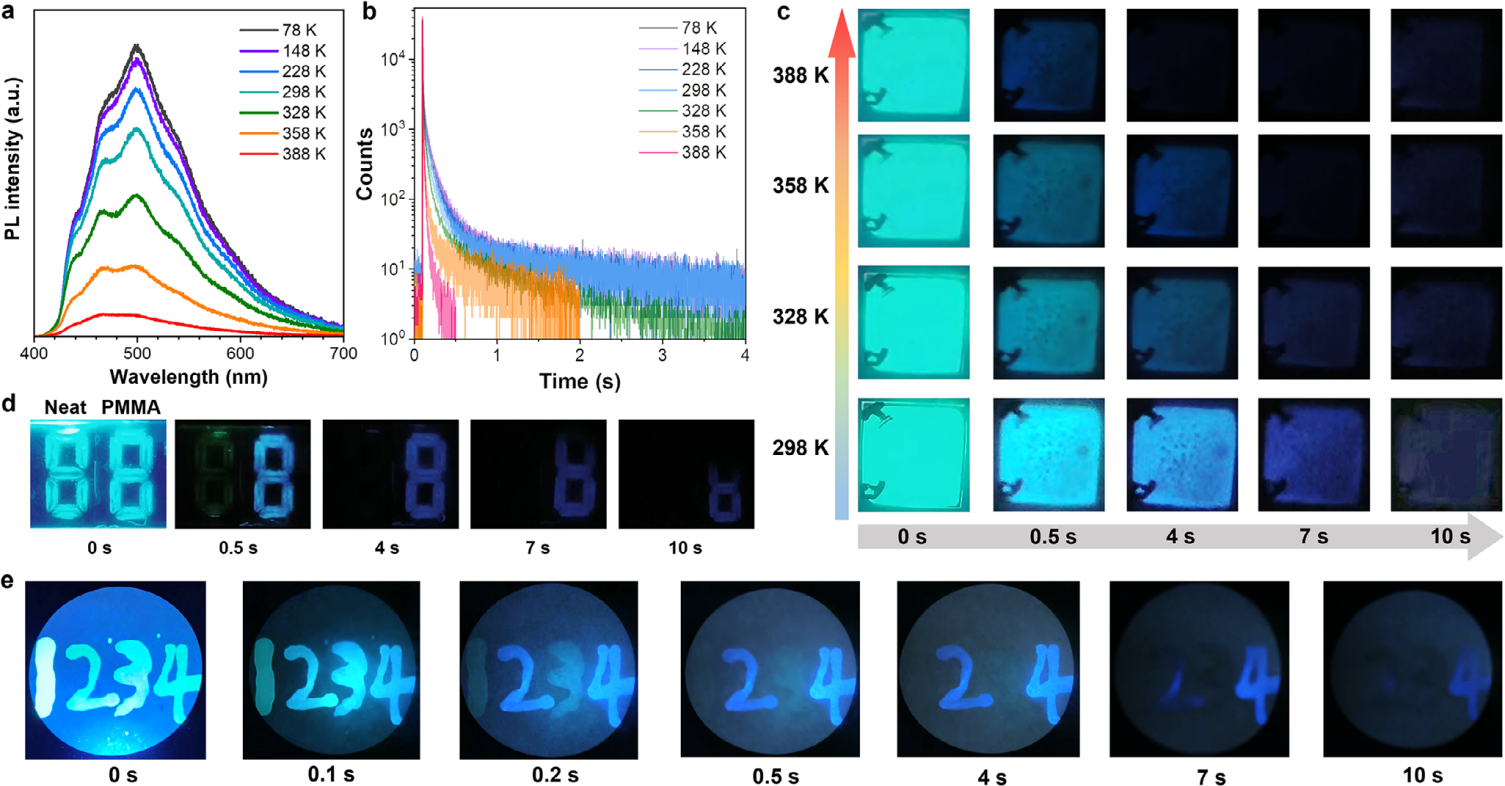
The photophysical behavior and information encryption display of dTC6‐BPSAF in doped polymer films. a) The phosphorescence spectra of doped PMMA films (1 wt.%) were excited at 330 nm. Delay time: 1 ms. b) The lifetime curves of the films (excited at 375 nm) recorded at their respective maximum phosphorescence peaks. c) Photographs of the films captured in air at 298 K before and after removal of the 365 nm UV light source. d) Information encryption photographs by different afterglow times of neat films (left) and PMMA films (1 wt.%) (right) designed based on dTC6‐BPSAF at 298K. e) The diagram of multiple anti‐counterfeiting applications. 1, 2, 3, 4 are constructed by dTC6‐BPSAF (1 wt.%): SBS, dTC6‐BPSAF (10 wt.%): PMMA, dTC6‐BPSAF (1 wt.%): PCL and dTC6‐BPSAF (1 wt.%): PMMA, respectively.

To investigate the RTP behaviors of dTC6‐BPSAF in rigid films, we systematically examined doped rigid polymer matrix films (Figure S16, Supporting Information). The phosphorescence properties of dTC6‐BPSAF in doped PMMA films were investigated. The phosphorescence spectral intensities (**Figure** **5**a) of the PMMA:1 wt.% dTC6‐BPSAF film retained 72% of their initial intensity at 298 K compared to those at 78 K, while maintaining stable structure‐resolved ^3^LE spectral profiles across temperatures. Notably, the films exhibited an ultralong phosphorescence lifetime of 1.16 s at room temperature (Figure 5b), with triplet exciton lifetimes remaining nearly constant (1.25 s at 78 K) over this temperature range. The persistent afterglow emission by the naked eye, lasting up to 10 s at 298K. By increasing the dTC6‐BPSAF doping concentration to 10 wt.% in PMMA, the phosphorescence lifetime can also remain 742 ms (Figure S17, Supporting Information), indicating that molecular motion and nonradiative transitions can also be effectively suppressed by PMMA at high doping concentrations. In addition, the atomic force microscopy (AFM) images (Figure S18, Supporting Information) display a smooth and homogeneous morphology with small root‐mean‐square (RMS) roughness values of 0.481 nm for 1 wt.% doped PMMA films and 0.546 nm for 10 wt.% doped PMMA films. It is free of particle aggregation or phase separation, suggesting both good film‐forming ability and good miscibility. Interestingly, the phosphorescence signal of dTC6‐BPSAF could still be observed, even at a high temperature of 388 K (Figure 5c). The outstanding high‐temperature tolerance of RTP could be attributed to the effective reduction in nonradiative decay, achieved by the rigidification of the molecular structure within a rigid polymer matrix. Specifically, the PMMA matrix effectively restricted the mobility of the alkoxy chains in dTC6‐BPSAF, thereby protecting triplet excitons from quenching and stabilizing the T_1_ state, which ultimately resulted in extended triplet‐state exciton lifetimes, enabling ultralong RTP emission. We also demonstrated a simple information anti‐counterfeiting application (Figure 5d) employing neat films and doped PMMA films (1 wt.%). The number “88” exhibited sky‐blue emission under UV light excitation. When the UV light was turned off, the portion of the number “8” written with the neat film disappeared due to its short‐lived fluorescence, while the remaining portion exhibited afterglow phosphorescence, transforming into a new number “8” after 10 s. Furthermore, we investigated the transient PL decay of dTC6‐BPSAF in doped PCL and SBS films (1 wt.%) with lifetimes of 180 and 49 ms, respectively (Figure S19, Supporting Information). The different afterglow emission lifetimes observed in these doped films make them promising candidates for advanced information storage and encryption applications utilizing time‐gated technology (Figure 5e).^[^ ^38^, ^39^, ^40^, ^41^ ^]^ Specific information can be captured sequentially: “1234” is obtained under UV irradiation; after turning off the UV light, “234” becomes recognizable at 0.2 s, “24” at 0.5 s, and “4” at 7 s, respectively. Thus, various numbers can be displayed at different times, contributing to dynamic information in encryption technology.

### 2.6 Photophysical Mechanisms in Various States

**Figure 6:**
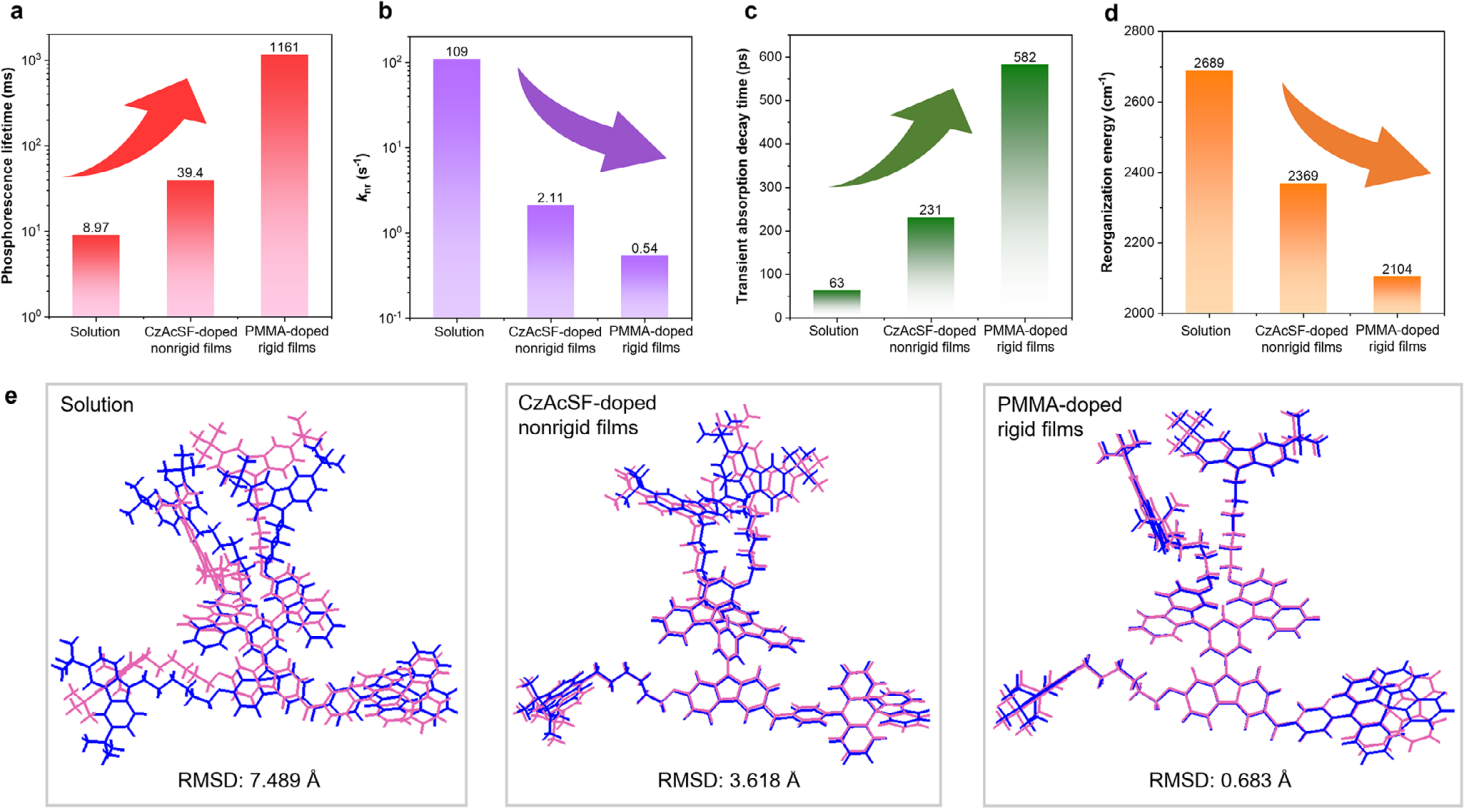
RTP mechanisms and nonradiative decay of dTC6‐BPSAF in solution, CzACSF‐doped nonrigid films, and PMMA‐doped rigid films. a) The phosphorescence lifetime plots of dTC6‐BPSAF in different states. b) The nonradiative transition rate (*k*_nr_) of dTC6‐BPSAF in different states. c) The transient absorption decay lifetime of dTC6‐BPSAF in different states. d) The calculated reorganization energy of dTC6‐BPSAF based on geometries optimized from S_0_ and T_1_ in different states. e) Overlaps of the optimized S_0_ and T_1_ geometries of dTC6‐BPSAF in different states. Color code: blue, S_0_ state; red, T_1_ state. The RMSD values of the atomic positions were calculated to evaluate the freedom of molecular motion reflected by structural differences between S_0_ and T_1_ states.

The RTP dendrimer dTC6‐BPSAF demonstrates distinct RTP performance in solution (in dilute toluene), nonrigid film (doped in host CzAcSF), and rigid film (doped in PMMA) environments, primarily due to significant differences in nonradiative transitions from T_1_ states across these three states. A detailed investigation of their photophysical properties holds critical implications for elucidating the underlying mechanisms of RTP behavior. Nonradiative decay is regulated by hierarchical rigidification, as matrices modulate intermolecular interactions to restrict motion. Accordingly, a gradual increase in the value of phosphorescence lifetime of dTC6‐BPSAF from in solution (8.97 ms) to in CzAcSF‐doped nonrigid films (39.4 ms) and PMMA‐doped rigid films (1161 ms) indicated an increase in molecular rigidity and stability of triplet excitons (**Figure** **6**a). This trend aligned with the experimental rate constants of nonradiative decay from the T_1_ states, *k*_nr_, where the solution exhibited the highest rate (*k*_nr_ = 109 s^−1^), followed by CzAcSF‐doped nonrigid films (*k*_nr_ = 2.11 s^−1^), while PMMA‐doped rigid films showed the lowest rate of 0.54 s^−1^ (Figure 6b). After the dTC6‐BPSAF dissolution, it has a high degree of freedom and is prone to molecular motions, resulting in the substantial dissipation of triplet excitons and the shortest lifetime, emphasizing the decisive role of nonradiative suppression in RTP performance. Moreover, the femtosecond transient absorption measurement of dTC6‐BPSAF in different states exhibits two decay time constants (Figures S20 and S21, Supporting Information), the first (faster, typically <10 ps) arises from exciton–phonon coupling (rapid energy transfer from excitons to molecular vibrations), while the second (slower, 10–1000 ps) is attributed to energy dissipation induced by molecular relaxation.^[^ ^42^ ^]^ The second decay time constants of the three decay curves in solution, CzAcSF‐doped nonrigid films, and PMMA‐doped rigid films are fitted to 582, 231, and 63 ps (Figure 6c), respectively. This decreasing trend directly reflects the modulation of molecular relaxation by hierarchical rigidification: In solution, dTC6‐BPSAF molecules possess high conformational freedom, allowing unconstrained rotation and vibration of both dendrons and the D‐A core. Such intense molecular motion prolongs the relaxation‐driven energy dissipation process, resulting in the largest time constant (582 ps). In nonrigid CzAcSF‐doped films, intermolecular aggregation partially restricts large‐amplitude motions (e.g., dendron entanglement reduces torsional freedom of the D‐A core), accelerating the relaxation‐induced dissipation and shortening the time constant to 231 ps. In rigid PMMA films, the polymer matrix forms a tight physical network with the alkyl chains of dTC6‐BPSAF, nearly quenching rotational and vibrational motions of the luminescent core. This strong confinement further limits nonradiative recombination or energy transfer to the surroundings, leading to the smallest time constant (63 ps).^[^ ^24^ ^]^ This correlation confirms that hierarchical rigidification modulates excited‐state dynamics by suppressing molecular relaxation, thereby preserving energy for radiative transitions.

Reorganization energy (λ_RE_) analysis quantitatively linked conformation changes induced by electronic excitation to energy dissipation. dTC6‐BPSAF in rigid films demonstrated the lowest λ_RE_ of 2104 cm^−1^, indicating minimal vibrational dissipation during the transition from T_1_ to S_0_ state. In contrast, dTC6‐ BPSAF in solution showed the highest λ_RE_ of 2698 cm^−1^, consistent with substantial nonradiative energy loss (Figure 6d). Vibrational mode decomposition revealed key motions contributing to λ_RE_, identifying vibrations relevant to T_1_‐S_0_ decay. The dominant normal mode (1763 cm^−1^) assigned to C═O stretching of the benzophenone acceptor contributed most significantly (Figure S22, Supporting Information). This mode's intensity was substantially lower in rigid film (253.5 cm^−1^) and nonrigid film (277.2 cm^−1^) than in solution (719.4 cm^−1^), demonstrating effective vibration suppression in solid states. Whereas solution‐phase conformational changes primarily involved torsion of the donor‐acceptor skeleton and rocking of alkyl‐carbazole dendrons, these motions were restricted in nonrigid/rigid matrices (Figure 6e). For instance, RMSD values from S_0_ and T_1_ decreased from 7.489 Å (solution) to 3.618 Å (nonrigid film) and further to 0.683 Å (rigid film), refining progressively restricted conformational freedom. In PMMA matrices, long‐chain entanglement with dTC6‐BPSAF's alkyl chains formed a physical network that restricts molecular motion and stabilizes *π*–*π* interactions. It can be inferred that the multiple intermolecular interactions between the RTP dendrimer and PMMA chains played a key role in suppressing nonradiative decay in these RTP films.

## 3 Conclusion

This study successfully resolves the long‐standing conflict between phosphorescent performance and material processability in organic RTP systems through an innovative multiscale confinement strategy. By integrating intramolecular flexible encapsulation with intermolecular rigid immobilization, we engineered dendronized donor‐acceptor molecules that simultaneously achieve environment‐adaptive luminescence and multifunctionality, overcoming the traditional “single‐environment” limitation. Key breakthroughs include oxygen‐resistant in‐solution RTP with a lifetime of ≈9 ms, efficient RTP films with PLQY of 72% and solution‐processed OLEDs with EQE of 17.2%, and the ultralong afterglow RTP materials with a lifetime of 1.16 and 10 s naked‐eye‐detectable emission. Crucially, the synergy of intramolecular through‐space interactions and multiscale confinement (from molecular aggregation to PMMA doping) suppresses nonradiative transitions across four temporal orders. This work not only establishes a universal design strategy for adaptive RTP materials but also pioneers a single‐material platform that unifies solution‐phase RTP, electroluminescence, and persistent afterglow—previously considered mutually exclusive functionalities. By systematically modulating intra‐ and intermolecular interactions, we provide foundational principles for next‐generation smart luminescent systems, with transformative potential in biomedical imaging, efficient optoelectronics, and advanced security technologies. Future efforts will focus on extending this strategy to other molecular architectures and scaling production for industrial adoption.

## Conflict of Interest

The authors declare no conflict of interest.

## Supporting information



Supporting Information

## Data Availability

The data that support the findings of this study are available in the supplementary material of this article.
