# Supplementary material for: Dendronized Encapsulation with Hierarchical Rigidification Enabling Robust Solution Room‐Temperature Phosphorescence, Efficient Electroluminescence and Ultralong Afterglow
Source: Adv Sci (Weinh). 2025 Aug 18;12(42):e11973. doi: 10.1002/advs.202511973 (PMC12622413; doi:10.1002/advs.202511973)
Supplement: Supplementary file 1 — Supporting Information [file ADVS-12-e11973-s001.pdf]

Supporting Information

Dendronized Encapsulation with Hierarchical Rigidification Enabling Robust Solution Room-Temperature Phosphorescence, Efficient Electroluminescence and Ultralong Afterglow

Chensen Li, Zhenchen Lou, Lianrui Hu, Guohua Xie, Song Zhang, Bo Xu,* Zheng Zhao,* Jacky W. Y. Lam* and Ben Zhong Tang**

Methods

Materials

Unless otherwise noted, all the chemicals and reagents were obtained from commercial sources (J&K, TCI, Meryer or Sigma Aldrich). The solvents for reactions were distilled and degassed before use. All reactions were carried out in an N_2 atmosphere with a dried Schlenk glassware or tube. ^1H and ^{13}C nuclear magnetic resonance (NMR) spectra were measured using deuterated $CDCl_3$, DMSO-d_6 as solvent. on a Bruker AV 500 spectrometer or a Bruker AVIII 400 spectrometer at room temperature. High-resolution mass spectra (HRMS) were obtained on a GCT premier CAB048 mass spectrometer operating in a MALDI-TOF mode. HPLC measurements were carried out on a Waters 2487 (600E) equipment with column of Poroshell 120 (EC-C18, 2.7 μm, 4.6 × 150 mm).

Characterization

UV-Vis absorption spectra were measured on a PerkinElmer Lambda 950 spectrophotometer. Photoluminescence (PL) spectra, transient PL decay spectra and photoluminescence quantum yields were performed on an Edinburgh FLS1000 fluorescence Spectrofluorometer. Cyclic voltammograms were measured in a solution of tetra-*n*-butylammonium hexafluorophosphate (nBu_4NPF_6, 0.1 M) in acetonitrile at a scan rate of 50 mV s^{-1}, using a platinum wire as the auxiliary electrode, a glass carbon disk as the working electrode, and Ag/Ag^+ as the reference electrode, standardized for the redox couple ferricenium/ferrocene (Fc/Fc^+) (HOMO = $-[E_{ox} + 4.8]$ eV, and LUMO = $-[E_{re} + 4.8]$ eV). E_{ox} and E_{re} represent the onset oxidation and reduction potentials relative to Fc/Fc^+, respectively. Thermogravimetric analysis (TGA) was carried out on TA TGA Q5000 under dry nitrogen at a heating rate of 10 °C min^{-1}. Thermal transition was investigated by TA DSC Q1000 under dry nitrogen at a heating rate of 10 °C min^{-1}.

Computational Details

The ground state geometries were optimized using the density functional theory (DFT) method with PBE0-D3 functional at the basis set level of 6-31G (d, p), and the excited-state geometries were optimized using the time-dependent DFT method with PBE0-D3 functional at the basis set level of 6-31G (d, p). The above calculations were performed using Gaussian16 package. The spin-orbital coupling constants were calculated based on PBE0/def2-SV(P) by using ORCA 5.0.2. The natural transition orbital analyses were analyzed with Multiwfn.

Device Fabrication and Characterization

The hole-injection material modified PEDOT:PSS (PEDOT:PSS-HJ-1, was purchased from Jinghang Optoelectronics (Shenzhen) Corporation and the electron transporting materials TmPyPB was obtained from Shanghai Xinrunsheng Corporation and used as received. The

ITO-coated glass with a sheet resistance of 15 Ω per square was used as the substrate. Before device fabrication, the ITO-coated glass substrate was precleaned and exposed to UV-ozone for 20 min. PEDOT:PSS was then spin-coated onto the clean ITO substrate as a hole-injection layer. Then, a solution of the emitter dissolved in chlorobenzene was spin-coated (10 mg/mL; 1000 rpm) to form a 40 nm-thick emissive layer and annealed at 50 °C for 30 min. Next, an exciton blocking layer of DPEPO with 10 nm thickness was vacuum deposited. Finally, a 50 nm-thick electron-transporting layer of TmPyPB was vacuum deposited, and a cathode composed of 1 nm-thick Liq and aluminum (100 nm) was deposited onto the substrate through shadow masking with a pressure of 10^{-6} Torr. The current density-voltage-luminance (*J-V-L*) characteristics of the devices were measured using a Keithley 2400 Source meter and a Spectroradiometer PhotoResearch PR735. The EL spectra were recorded simultaneously with PR735. The EQE values were calculated from the luminance, current density, and electroluminescence spectrum according to previously reported methods. All measurements were performed at room temperature under ambient conditions after device encapsulation with UV curable resin.

Synthesis

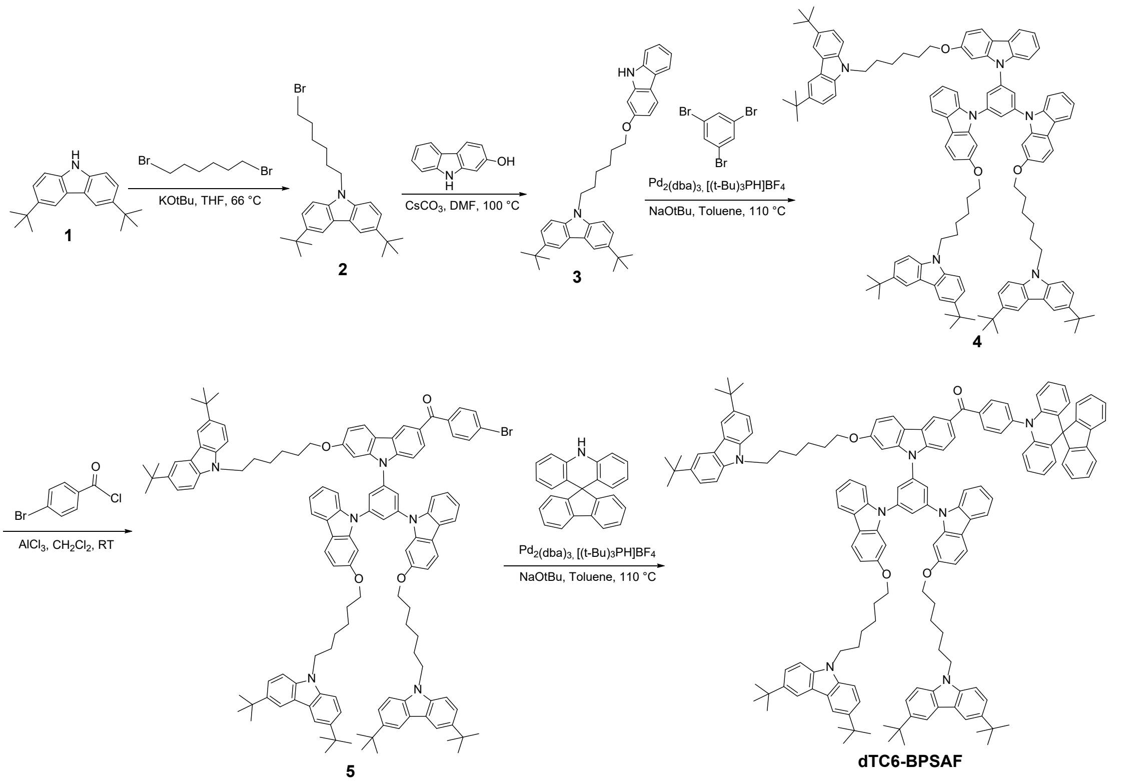

Scheme S1. The synthesis routes of dTC6-BPSAF.

9-(6-bromohexyl)-3,6-di-*tert*-butyl-9*H*-carbazole (2)

To a solution of compound 3,6-di-*tert*-butyl-9*H*-carbazole (1)[24] (1.4 g, 5 mmol) in dry THF (10 mL) was added KO^{t}Bu (0.706 g, 6.3 mmol) in several portions. The resulting solution was stirred for 10 min at room temperature, and then added dropwise to a refluxing solution of 1,6-dibromohexane (4.5 mL, 29.2 mmol) in THF (10 mL). After refluxing for 24 h, the reaction mixture was cooled to room temperature and quenched by adding several drops of water to destroy the excessive KO^{t}Bu. The THF solvent and excess 1,6-dibromohexane were removed

by rotatory evaporation and vacuum distillation, respectively. The residual product was dissolved in CH_2Cl_2, washed with water for three times, the organic layer was dried over anhydrous Na_2SO_4, filtered and concentrated for flash column chromatography on silica using CH_2Cl_2/petroleum ether (1/10 v/v), affording product as a white solid (1.76 g 80% yield). ^{1}H NMR (400 MHz, $CDCl_3$) δ 8.15 (d, *J* = 1.7 Hz, 1H), 7.55 (dd, *J* = 8.6, 1.9 Hz, 1H), 7.33 (d, *J* = 8.6 Hz, 1H), 4.28 (t, *J* = 7.1 Hz, 1H), 3.39 (t, *J* = 6.7 Hz, 1H), 1.95 – 1.77 (m, 2H), 1.51 (s, 9H), 1.49 – 1.39 (m, 2H). ^{13}C NMR (101 MHz, $CDCl_3$) δ 140.90, 138.34, 122.67, 122.07, 115.70, 107.37, 42.34, 34.07, 33.23, 32.00, 31.50, 28.40, 27.37, 25.91. MALDI-MS$^+$: m/z: calculated for $C_{26}H_{36}BrN$: 441.2031 $[M]^+$; found: 441.2026 $[M]^+$.

9-(6-((9*H*-carbazol-2-yl)oxy)hexyl)-3,6-di-*tert*-butyl-9*H*-carbazole (3)

A mixture of 9-(6-bromohexyl)-3,6-di-*tert*-butyl-9*H*-carbazole (2) (441 mg, 1.0 mmol), 9H-carbazol-2-ol (183 mg, 1.0 mmol) and Cs_2CO_3 (391 mg, 1.2 mmol) in *N,N*-dimethylformamide (10 mL) solution was heated at 100 °C under nitrogen for 24 h. After cooling, the mixture was poured into water (200 mL). The crude product was filtered and purified by silica gel column chromatography, eluent CH_2Cl_2/petroleum ether (1/2 v/v). The product was recrystallized from ethyl acetate to give product as a white powder (462 mg, 85%). ^{1}H NMR (400 MHz, $CDCl_3$) δ 8.31 (d, *J* = 7.7 Hz, 1H), 8.11 (d, *J* = 1.6 Hz, 2H), 8.06 (s, 1H), 7.50 (dd, *J* = 8.6, 1.9 Hz, 2H), 7.43 – 7.35 (m, 2H), 7.34 – 7.29 (m, 3H), 7.23 (m, *J* = 8.1, 6.4, 1.8 Hz, 1H), 7.03 (d, *J* = 8.0 Hz, 1H), 6.65 (d, *J* = 7.9 Hz, 1H), 4.34 – 4.14 (m, 4H), 2.03 – 1.88 (m, 4H), 1.74 – 1.62 (m, 2H), 1.54 (dd, *J* = 15.2, 7.0 Hz, 2H), 1.47 (s, 18H). ^{13}C NMR (101 MHz, $CDCl_3$) δ 140.80, 138.34, 126.05, 124.24, 122.63, 122.35, 122.01, 119.00, 115.63, 109.28, 107.38, 100.41, 67.07, 42.40, 34.03, 31.45, 28.71, 26.50, 25.55. MALDI-MS$^+$: m/z: calculated for $C_{38}H_{44}N_2O$: 544.3454 $[M]^+$; found: 544.3412 $[M]^+$.

1,3,5-tris(2-((6-(3,6-di-*tert*-butyl-9H-carbazol-9-yl)hexyl)oxy)-9*H*-carbazol-9-yl)benzene (4)

A mixture of compound 9-(6-((9*H*-carbazol-2-yl)oxy)hexyl)-3,6-di-*tert*-butyl-9*H*-carbazole (3) (554 mg, 1 mmol), 1,3,5-tribromobenzene (105 mg, 0.33 mmol), tris(dibenzylideneacetone)dipalladium ($Pd_2(dba)_3$) (38 mg, 0.037 mmol), $(t\text{-}Bu)_3PHBF_4$ (21 mg, 0.073 mol) and sodium tert-butoxide (NaO^tBu) (210 mg, 2.19 mmol) in toluene (20 mL) was stirred at 110 °C under argon for 24 h. After cooling to room temperature, the mixture was washed with brine and the organic phase was separated and dried with anhydrous sodium sulfate. After filtration and removal of the solvent, the product mixture was applied to a silica gel column using hexane/dichloromethane (3/1 v/v) as eluent to give the crude product as white power. The powder was further crystallized from a mixture of hexane and CH_2Cl_2 to afford the pure product (382 mg, 82% yield) as a white powder. ^{1}H NMR (400 MHz, $CDCl_3$) δ 8.53 (d, *J* = 7.7 Hz, 1H), 8.24 (d, *J* = 1.6 Hz, 2H), 8.06 (s, 1H), 7.75 (d, *J* = 8.2 Hz, 1H), 7.60 (dd, *J* = 8.6, 1.9 Hz, 2H), 7.53 (dd, *J* = 11.4, 4.1 Hz, 1H), 7.48 – 7.35 (m, 5H), 6.82 (d, *J* = 8.0 Hz, 1H), 4.37 (t, *J* = 7.0 Hz, 2H), 4.30 (t, *J* = 6.2 Hz, 2H), 2.04 (dt, *J* = 14.6, 9.4 Hz, 4H), 1.77 (dd, *J* = 14.7, 7.8 Hz, 2H), 1.65 (s, 2H), 1.58 (s, 18H). ^{13}C NMR (101 MHz, $CDCl_3$) δ 155.91, 141.85, 141.54, 140.87, 139.69, 139.08, 127.29, 125.49, 123.80, 123.51, 123.43, 123.38, 122.78, 121.02, 116.37, 113.14, 109.19, 108.13, 102.52, 68.01, 43.11, 34.78, 32.22, 31.74, 29.86, 29.42, 29.31, 27.23, 26.29, 22.81, 14.31. MALDI-MS$^+$: m/z: calculated for $C_{120}H_{132}N_6O_3$: 1705.0361 $[M]^+$; found: 1706.0444 $[M+H]^+$.

(9-(3,5-bis(2-((6-(3,6-di-*tert*-butyl-9*H*-carbazol-9-yl)hexyl)oxy)-9*H*-carbazol-9-yl)phenyl)-7-((6-(3,6-di-*tert*-butyl-9*H*-carbazol-9-yl)hexyl)oxy)-9*H*-carbazol-3-yl)(4-bromophenyl)methanone (5)

Aluminum trichloride (112 mg, 0.84 mmol) was added to a stirred solution of 1,3,5-tris(2-((6-(3,6-di-*tert*-butyl-9H-carbazol-9-yl)hexyl)oxy)-9*H*-carbazol-9-yl)benzene (4) (1.71 g, 1 mmol) and 4-bromobenzoyl chloride (200 mg, 0.91 mmol) in dehydrated dichloromethane (50 mL) in ice bath for 15 minutes. Then the reaction mixture was warmed to room temperature and stirred for 3 h. The reaction was quenched with ice water and hydrochloric acid (15 mL, 2:1 v/v), and extracted with dichloromethane several times. The combined organic layers were washed twice with water, and then dried over anhydrous $MgSO_4$. After filtration and solvent evaporation under reduced pressure, the residue was purified by column chromatography on silica gel (dichloromethane/petroleum ether) to afford a green solid in 48% yield. ^{1}H NMR (400 MHz, $CDCl_3$) δ 8.92 – 8.70 (m, 4H), 8.57 – 8.46 (m, 5H), 8.45 – 8.38 (m, 1H), 8.30 (d, *J* = 28.7 Hz, 2H), 8.17 – 8.12 (m, 1H), 8.05 – 7.93 (m, 3H), 7.88 – 7.72 (m, 13H), 7.61 (dt, *J* = 16.6, 14.2 Hz, 14H), 7.45 (ddd, *J* = 26.9, 13.8, 5.6 Hz, 2H), 6.92 (s, 2H), 4.57 – 4.23 (m, 12H), 2.17 (m, 10H), 2.02 (m, 2H), 1.95 – 1.86 (m, 6H), 1.82 (s, 54H), 1.73 (m, 6H). ^{13}C NMR (101 MHz, $CDCl_3$) ^{13}C NMR (101 MHz, $CDCl_3$) δ 198.87, 195.05, 161.91, 155.53, 155.36, 145.07, 142.20, 141.47, 141.39, 141.35, 141.09, 140.86, 140.46, 140.09, 139.79, 139.34, 139.26, 138.72, 138.31, 137.35, 136.91, 136.65, 135.58, 131.81, 131.57, 131.10, 130.40, 128.28, 126.91, 125.87, 125.15, 125.05, 124.02, 123.25, 123.14, 123.00, 122.76, 122.47, 122.23, 122.08, 121.38, 120.67, 119.86, 119.16, 118.22, 116.08, 115.95, 115.72, 113.07, 112.89, 112.81, 112.63, 112.04, 111.75, 108.67, 108.25, 107.80, 102.12, 67.49, 60.00, 53.04, 42.57, 34.40, 34.35, 34.26, 31.84, 31.79, 31.76, 31.67, 31.63, 31.58, 31.38, 28.91, 28.79, 27.04, 26.78, 26.67, 26.13, 25.88, 25.58, 22.45, 22.27, 20.63, 13.94. MALDI-MS$^+$: m/z: calculated for $C_{127}H_{135}BrN_6O_4$: 1886.9728 [M]$^+$; found: 1887.9772 [M+H]$^+$.

(4-(10H-spiro[acridine-9,9'-fluoren]-10-yl)phenyl)(9-(3,5-bis(2-((6-(3,6-di-tert-butyl-9H-carbazol-9-yl)hexyl)oxy)-9H-carbazol-9-yl)phenyl)-7-((6-(3,6-di-tert-butyl-9H-carbazol-9-yl)hexyl)oxy)-9H-carbazol-3-yl)methanone (dTC6-BPSAF)

A mixture of compound (9-(3,5-bis(2-((6-(3,6-di-*tert*-butyl-9*H*-carbazol-9-yl)hexyl)oxy)-9*H*-carbazol-9-yl)phenyl)-7-((6-(3,6-di-*tert*-butyl-9*H*-carbazol-9-yl)hexyl)oxy)-9*H*-carbazol-3-yl)(4-bromophenyl)methanone (5) (1.89 g, 1 mmol), 10H-spiro[acridine-9,9'-fluorene] (332 mg, 1 mmol), tris(dibenzylideneacetone)dipalladium ($Pd_2(dba)_3$) (38 mg, 0.037 mmol), (t-Bu)$_3$PHBF$_4$ (21 mg, 0.073 mol) and sodium tert-butoxide (NaO^{t}Bu) (210 mg, 2.19 mmol) in toluene (20 mL) was stirred at 110 °C under argon for 24 h. After cooling to room temperature, the mixture was washed with brine and the organic phase was separated and dried with anhydrous sodium sulfate. After filtration and removal of the solvent, the product mixture was applied to a silica gel column using hexane/dichloromethane (1/1 v/v) as eluent to give the crude product as white power. The powder was further crystallized from a mixture of hexane and CH_2Cl_2 to afford the pure product (1.7 g, 79% yield) as a green powder. ^{1}H NMR (400 MHz, $CDCl_3$) δ 8.79 – 8.59 (m, 4H), 8.51 – 8.34 (m, 6H), 8.31 (d, *J* = 8.1 Hz, 1H), 8.22 (d, *J* = 10.3 Hz, 3H), 8.03 – 7.87 (m, 7H), 7.86 – 7.80 (m, 1H), 7.80 – 7.62 (m, 13H), 7.62 – 7.47 (m, 15H), 7.46 (d, *J* = 7.0 Hz, 2H), 7.41 – 7.29 (m, 1H), 7.21 (dd, *J* = 16.5, 8.3 Hz, 1H), 6.85 (ddd, *J* = 31.4, 18.3, 9.3 Hz, 5H), 6.75 – 6.63 (m, 3H), 4.44 (dd, *J* = 24.1, 17.5 Hz, 12H), 2.15 (s,

10H), 1.89 (dd, J = 31.5, 25.8 Hz, 8H), 1.74 (s, 54H), 1.63 (s, 6H). ^{13}C NMR (101 MHz, $CDCl_3$) δ 195.41, 155.96, 155.45, 145.46, 142.25, 141.33, 141.06, 140.56, 140.39, 139.77, 139.18, 138.84, 138.64, 138.24, 137.81, 135.63, 133.04, 131.36, 131.03, 128.04, 127.66, 127.32, 126.97, 126.84, 125.32, 125.12, 125.00, 124.80, 124.70, 123.21, 122.94, 122.67, 122.35, 122.14, 121.49, 120.74, 119.82, 119.60, 118.15, 116.05, 115.90, 114.09, 112.80, 112.70, 108.72, 108.20, 107.72, 102.07, 67.49, 56.39, 53.04, 42.57, 34.39, 34.31, 31.78, 31.73, 31.60, 31.27, 29.41, 28.91, 28.83, 26.96, 26.72, 26.27, 26.04, 25.82, 22.35, 13.88. MALDI-MS$^+$: m/z: calculated for $C_{152}H_{151}N_7O_4$: 2138.1828 $[M]^+$; found: 2139.0918 $[M+H]^+$. Anal. calcd for $C_{152}H_{151}N_7O_4$ (%): C, 85.31; H, 7.11; N, 4.58; O, 2.99. found: C, 85.29; H, 7.07; N, 4.55; O, 2.96.

Thermology properties

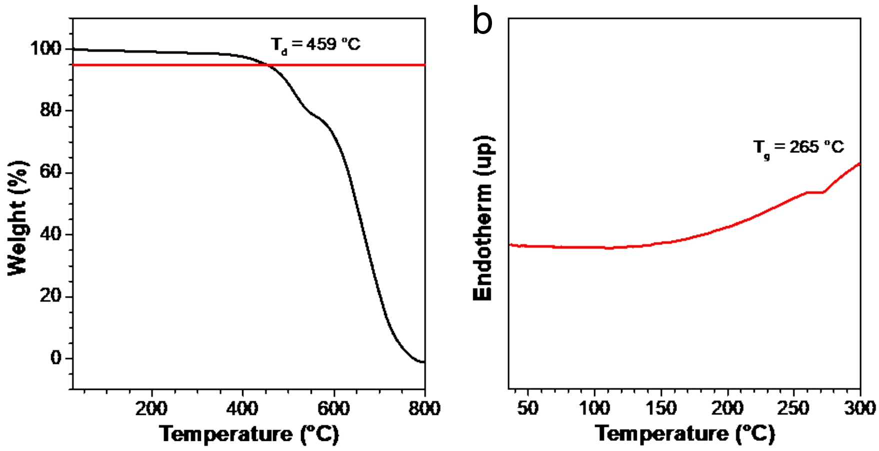

Figure S1. TGA measurements (a) and DSC traces (b) of dTC6-BPSAF recorded at a heating rate of 10 °C/min under nitrogen atmosphere.

Electrochemical properties

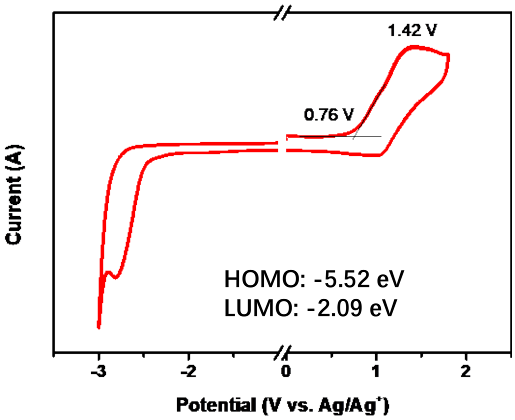

Figure S2. Cyclic voltammetry curves of dTC6-BPSAF.

Theoretical calculation

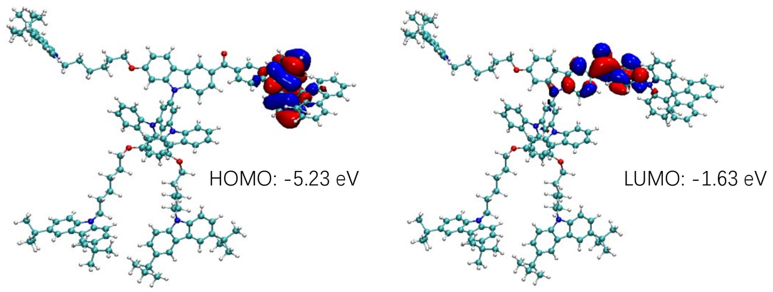

Figure S3. The HOMOs and LUMOs distributions and energy levels of dTC6-BPSAF at the optimized S_0 geometries.

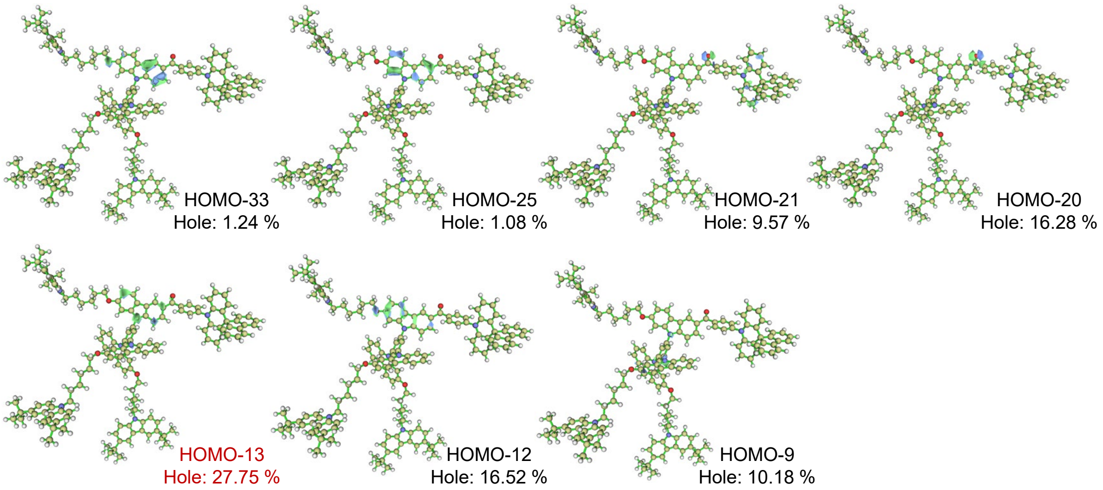

Figure S4. The contributions of HOMOs to the hole in the T_1 state of dTC6-BPSAF.

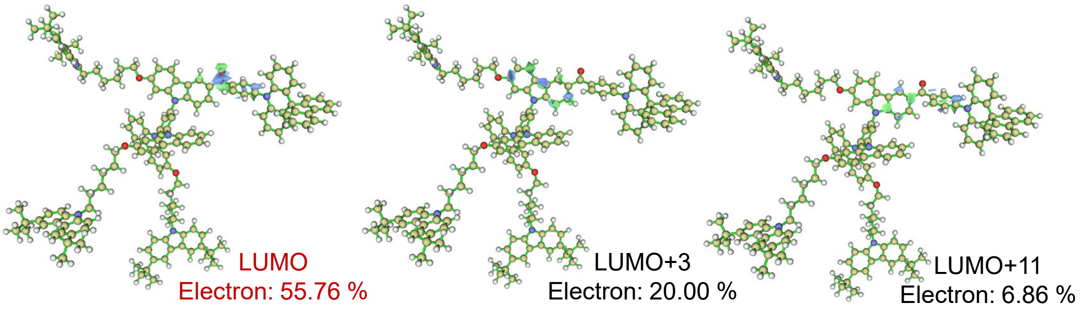

Figure S5. The contributions of LUMOs to the electron in the T_1 state of dTC6-BPSAF.

Photophysical properties in solution

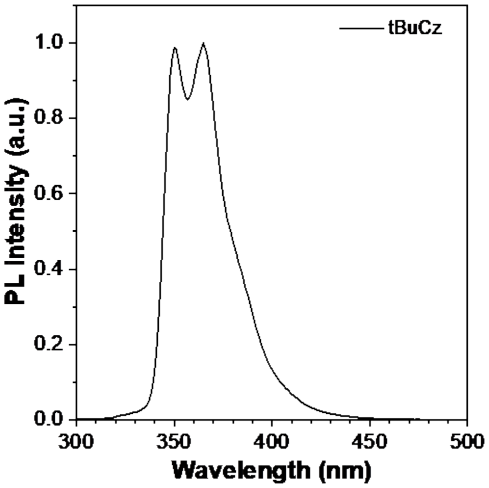

Figure S6. The PL spectra of 3,6-di-tert-butylcarbazole (tBuCz) in dilute toluene.

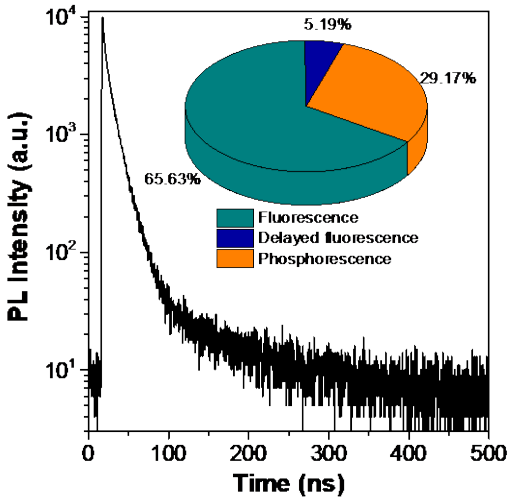

Figure S7. The PL transient decay in microsecond range of dTC6-BPSAF in toluene solution.

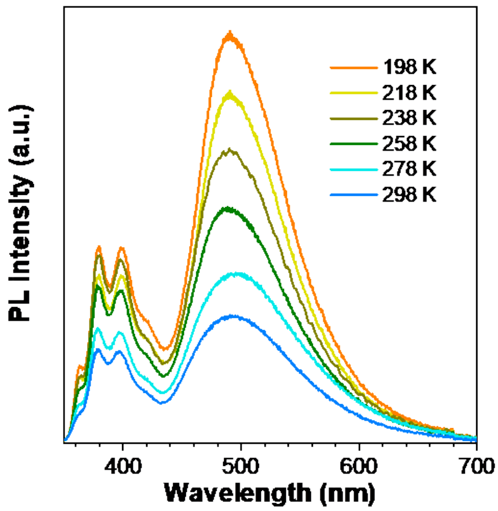

Figure S8. The PL spectra of dTC6-BPSAF in toluene at different temperature.

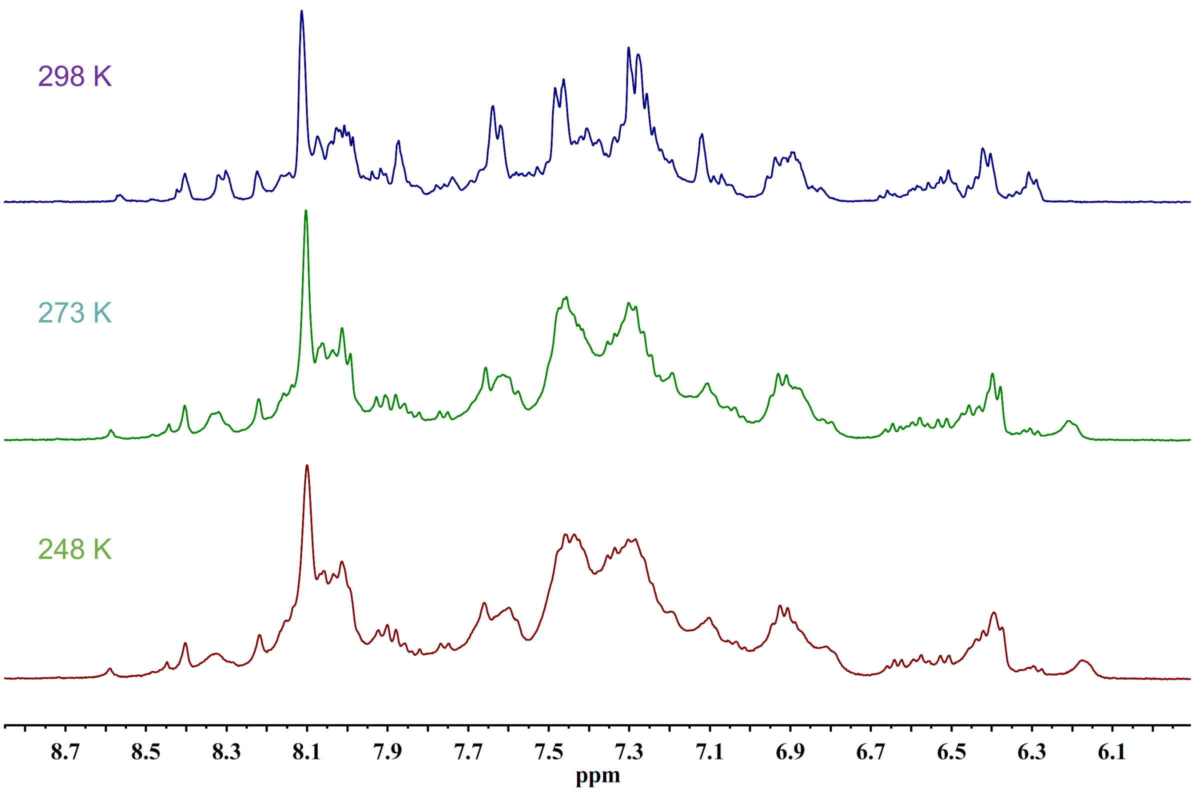

Figure S9. The ^1H NMR spectra of dTC6-BPSAF measured in CD_2Cl_2 from 248 K to 298 K.

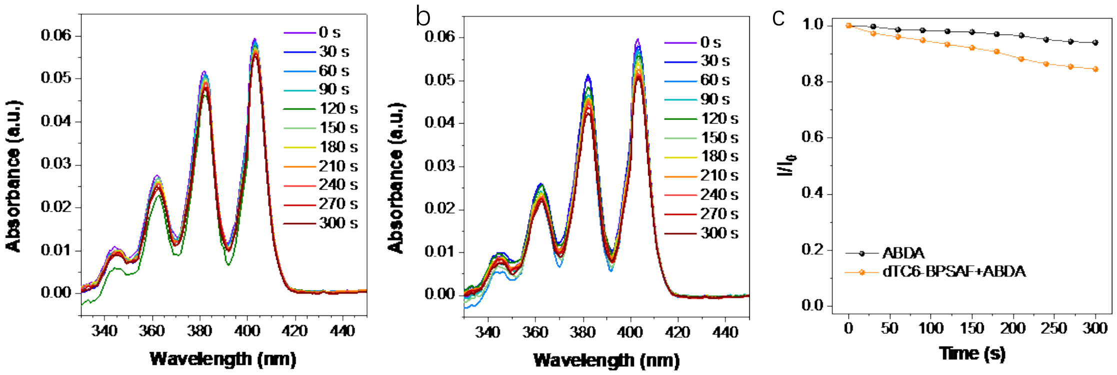

Figure S10. The UV-vis spectra of ABDA in the presence of (a) blank control, dTC6-BPSAF (b). The ROS generation under UV irradiation were measured, employing 9,10-anthracenediyl-bis(methylene)dimalonic acid (ABDA) as a singlet oxygen ($^1O_2$) indicator. (c) Access of $^1O_2$ generation with the decomposition of ABDA for dTC6-BPSAF under light irradiation.

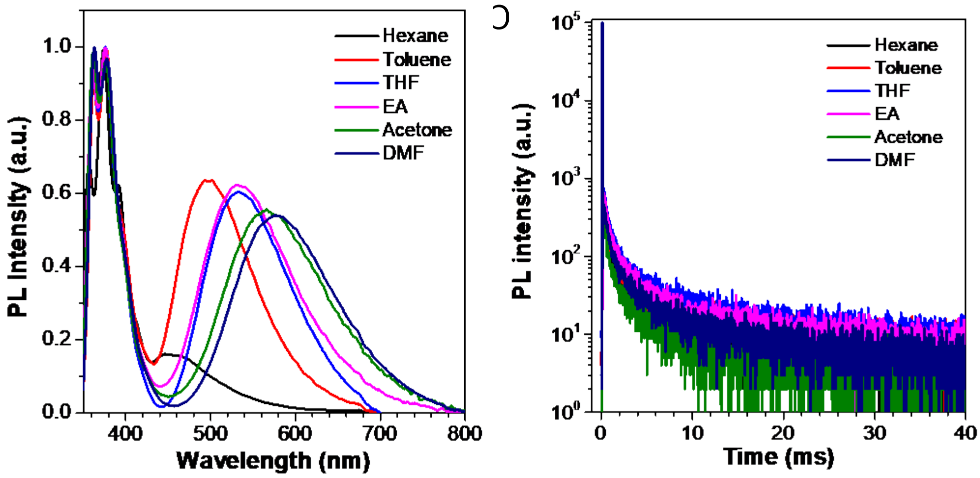

Figure S11. The PL spectra of dTC6-BPSAF (a) and their transient PL decay (b) in different solvents.

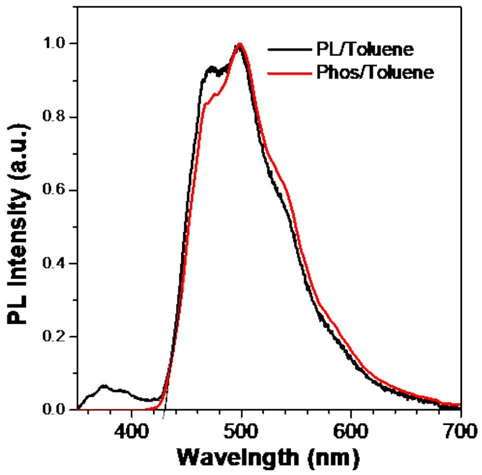

Figure S12. The PL and Phos spectra of dTC6-BPSAF at 78 K in toluene.

Table S1. Summary of the detailed photophysical parameters of the investigated dendrimers in toluene and water/THF mixtures.

	dTC6-BPSAF (Toluene)	10% water - dTC6-BPSAF	90% water-dTC6-BPSAF
τ_F (ns)	7.07	1.68	6.23
F [%]	65.7	79.7	45.7
τ_{DF} (ns)	99.5	78.9	388
DF [%]	5.06	6.6	19.4
τ_P (ms)	8.97	3.18	28.0
RTP [%]	29.2	13.7	34.9
φ_{PL} (%)	9	1	15
φ_F (%)	5.9	0.80	6.85
φ_{DF} (%)	0.46	0.066	2.91
φ_P (%)	2.6	0.14	5.24

k_F (10^6 s^{-1})	8.36	4.74	11.0
k_{RISC} (10^6 s^{-1})	0.643	0.11	0.26
k_P (s^{-1})	2.93	0.43	1.87
k_{nr} (10^2 s^{-1})	1.09	3.14	0.34
k_{ISC} (10^6 s^{-1})	4.36	1.21	13.1

Photophysical properties in non-rigid films

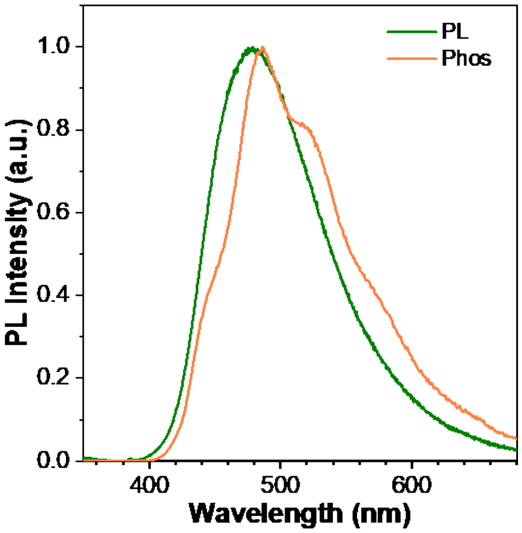

Figure S13. The PL and Phos spectra of dTC6-BPSAF in doped CzAcSF films.

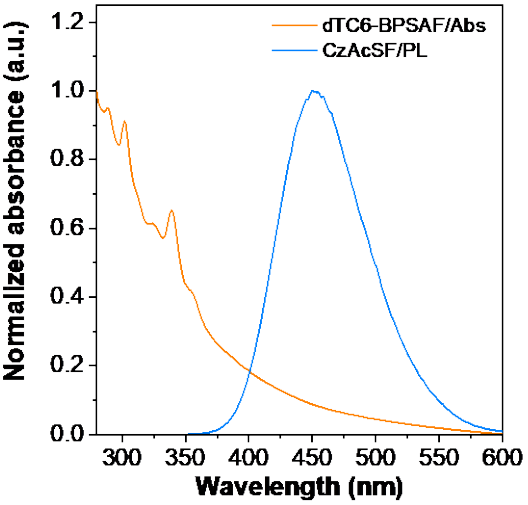

Figure S14. The PL spectra of CzAcSF and UV-vis absorption spectra of dTC6-BPSAF.

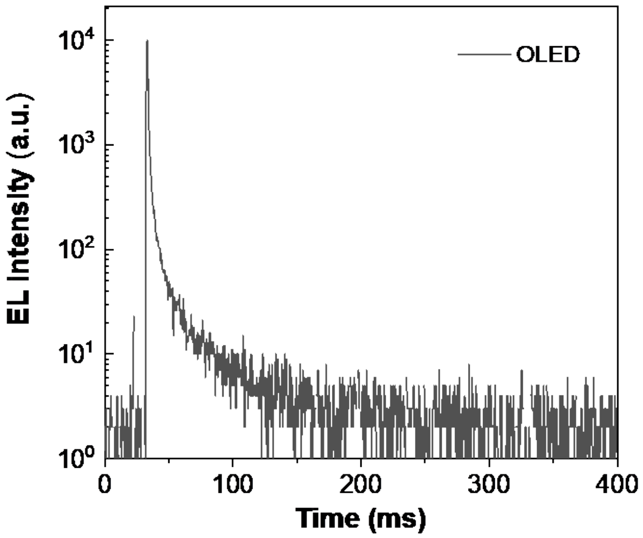

Figure S15. The EL transient decay spectra the solution-processed OLEDs at 6 V based on CzAcSF: dTC6-BPSAF (90: 10 wt%).

Table S2. Summary of photophysical data for dTC6-BPSAF in neat films and host doped films.

	λ_{PL}[a] (nm)	PLQY[b] (%)	ΔE_{ST}[c] (eV)	PF[d] (ns/%)	DF[e] (μs/%)	Ph[f] (ms/%)	k_{ISC}[g] (10^7 s^{-1})	k_{RISC}[h] (10^4 s^{-1})	k_F[i] (10^6 s^{-1})	k_P[j] (10^0 s^{-1})	k_{nr}[k] (10^1 s^{-1})
**dTC6-BPSAF**	492	31	0.03	9.23/22.6	6.9/22.0	39.4/55.4	2.60	2.15	7.59	4.36	2.10
**Doped dTC6-BPSAF**	478	72	0.02	16.3/20.0	3.8/21.1	37.1/58.9	3.53	9.18	8.83	11.4	1.55

[a]The peak values of PL spectra. [b]Absolute PL quantum yield determined by a calibrated integrating sphere in degassed conditions; error ± 2%. [c]Singlet-triplet energy gap in neat films and doped films at 78K. [d]The fluorescence lifetime and ratio component. [e]The delayed fluorescence lifetime and ratio component. [f]The phosphorescence lifetime and ratio component. [g]The rate constant of intersystem crossing. [h]The rate constant of reversed intersystem crossing. [i]The radiative decay rate of singlet exciton. [j]The radiative decay rate of triplet exciton. [k]The nonradiative decay rate of triplet exciton. The rate constants are calculated using the following equations: $k_{ISC} = (\varphi_{P+}\varphi_{DF})/\tau_F$, $k_{ISC} = (\varphi_{F+}\varphi_{DF})/(\tau_{DF}\times(1-\varphi_{DF}))$, $k_F = \varphi_F/\tau_F$, $k_P = \varphi_P/\tau_P$, $k_{nr} = (1-\varphi_P)/\tau_P$.

Device fabrication and characterization

Table S3. EL properties of the solution-processed devices based on CzAcSF: 10%/20% dTC6-BPSAF.

	V_{on} (V)	L_{max} (cd/m^2)	CE_{max} (cd/A)	PE_{max} (lm/W)	EQE_{max} (%)	EL peak (nm)	CIE (x,y)
10% dTC6-BPSAF	5.5	757	38.6	20.2	17.9	492	0.18. 0.36
20% dTC6-BPSAF	5.5	983	32.2	16.8	12.3	492	0.19. 0.36

Photophysical properties in rigid films

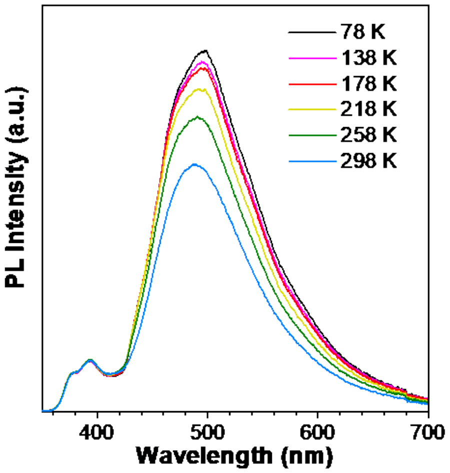

Figure S16. Temperature-dependent PL spectra of dTC6-BPSAF in doped PMMA films (1 wt%) at 78 K.

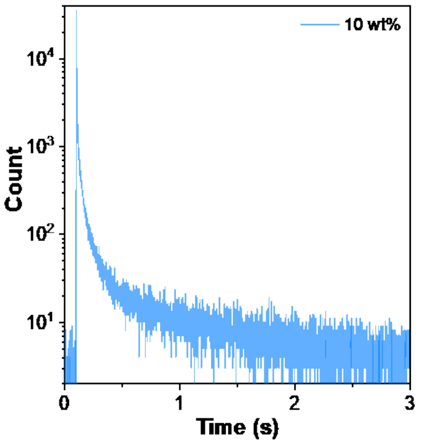

Figure S17. The PL transient decay of dTC6-BPSAF in doped PMMA (10 wt%).

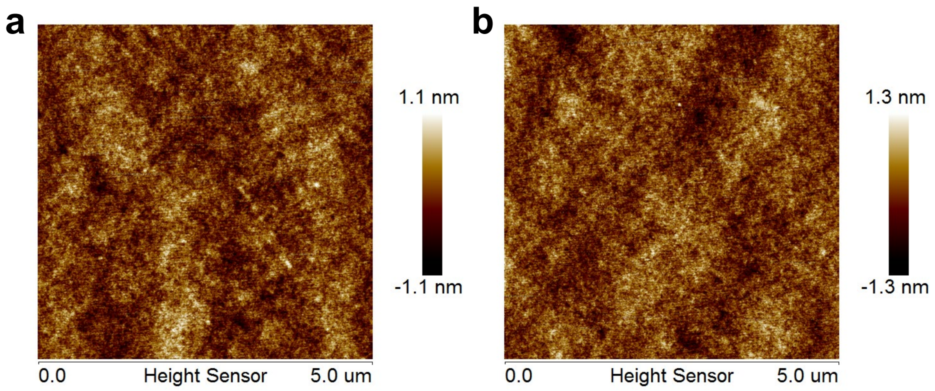

Figure S18. The atomic force microscopy (AFM) topographic images of the dTC6-BPSAF in doped PMMA with 1 wt% (**a**) and 10 wt% (**b**).

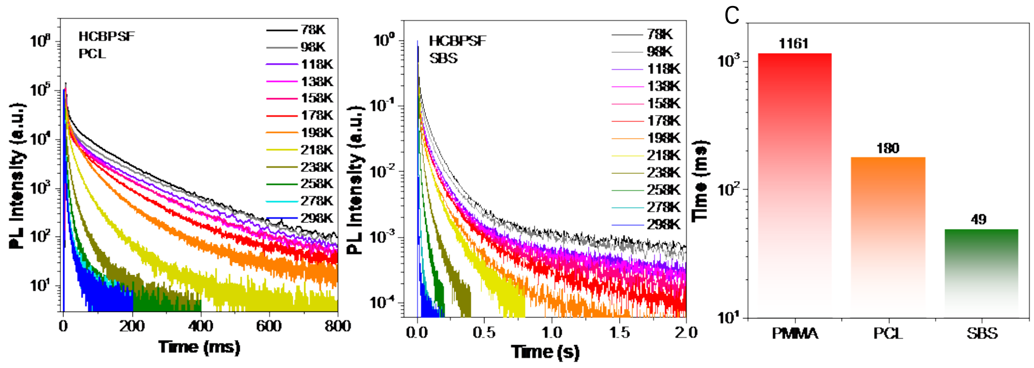

Figure S19. The temperature-dependent PL transient decay of dTC6-BPSAF in doped PCL (1 wt%) (**a**) and SBS (1 wt%) (**b**) films. (**c**) The plots of phosphorescence lifetimes of dTC6-BPSAF in PMMA, PCL and SBS doped films at 298 K. The hardness of PMMA, PCL, and SBS are about 77, 53, and 47 (Shore -D), respectively.[1-3]

Photophysical mechanisms in different states

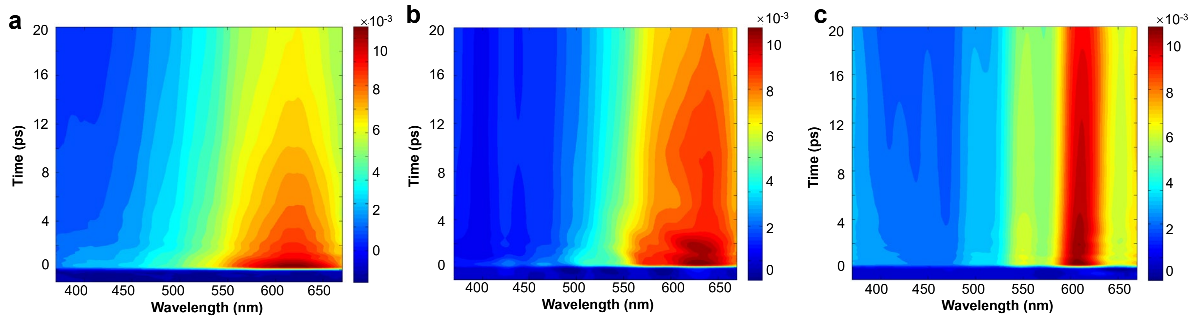

Figure S20. Two-dimensional transient absorption spectral map of the dTC6-BPSAF between 0 and 20 ps with the pump at 330 nm in toluene, host doped film, and PMMA doped film.

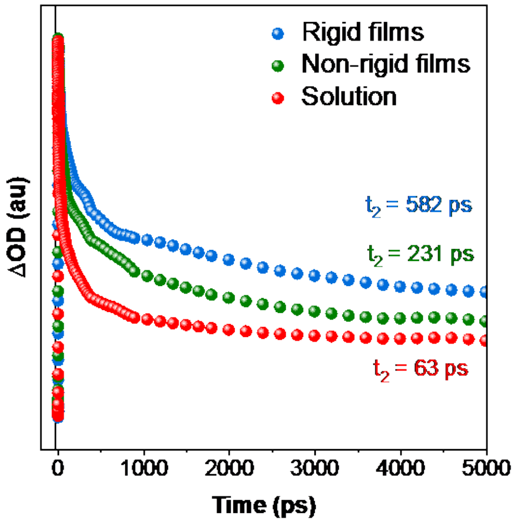

Figure S21. Kinetic traces probed at 610 nm and the corresponding fit. ΔOD, changes in absorbance.

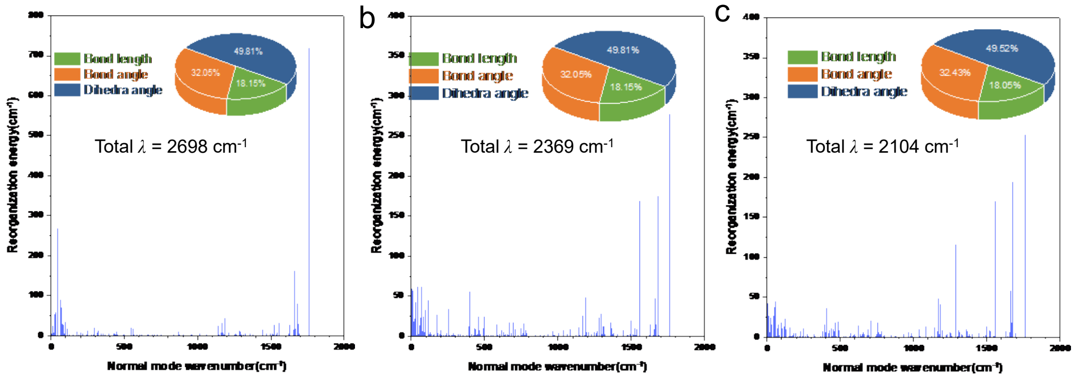

Figure S22. Plots of reorganization energy (λ) vs normal mode wavenumber of dTC6-BPSAF in toluene, host doped film, and PMMA doped film. Inset: Proportions of bond length, bond angle, and dihedral angle contributed to total reorganization energy.

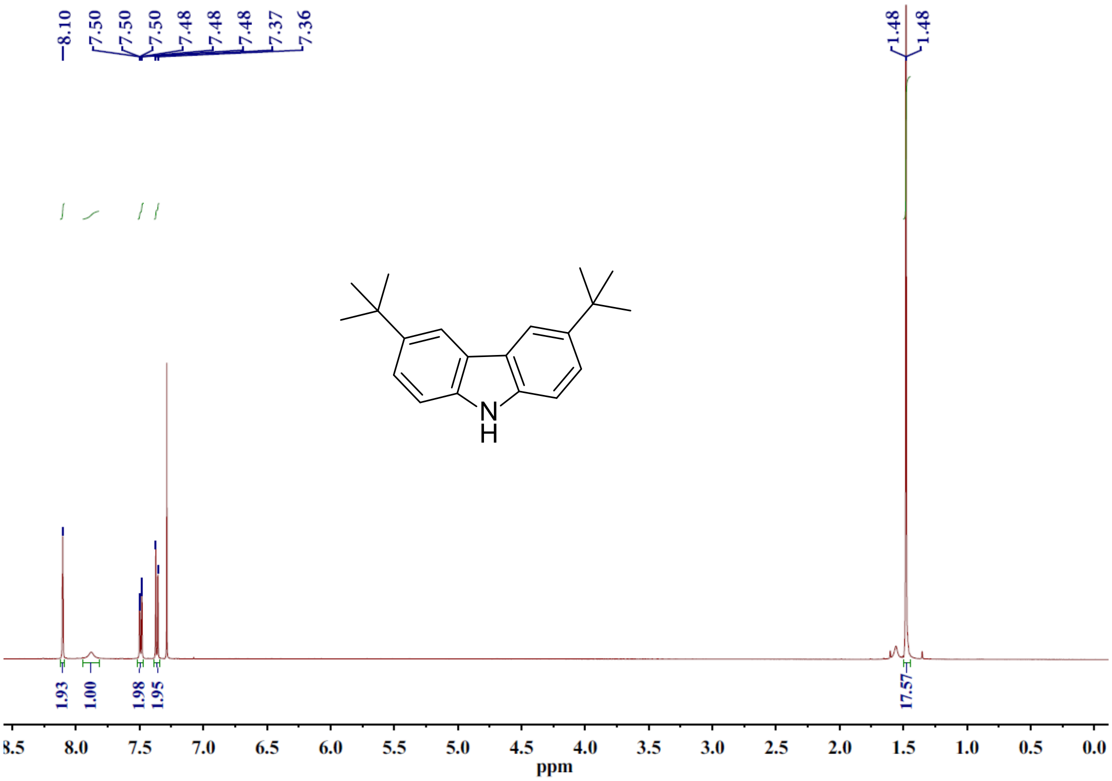

Figure S23. ^{1}H NMR spectra of 1.

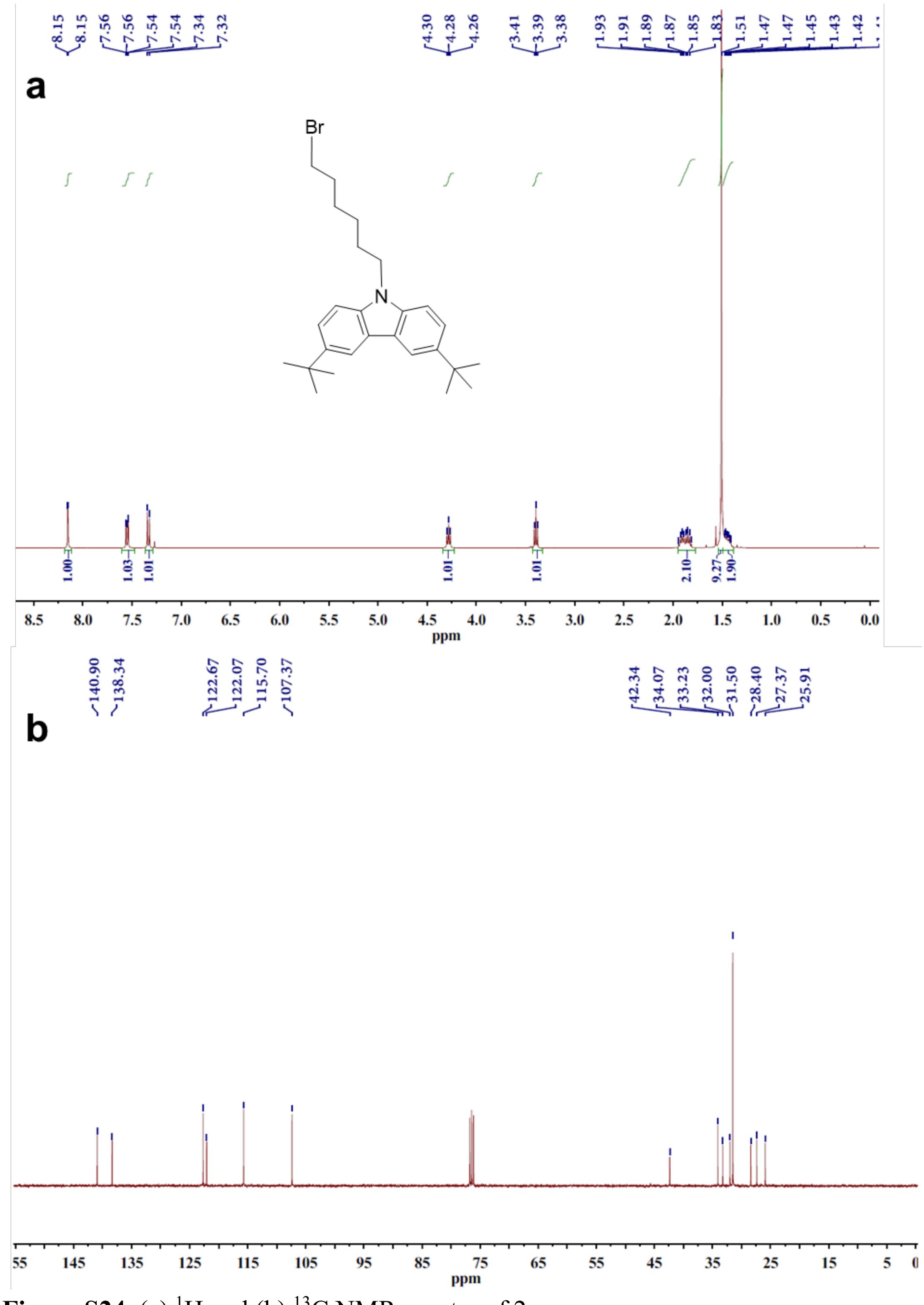

Figure S24. (a) ^{1}H and (b) ^{13}C NMR spectra of 2.

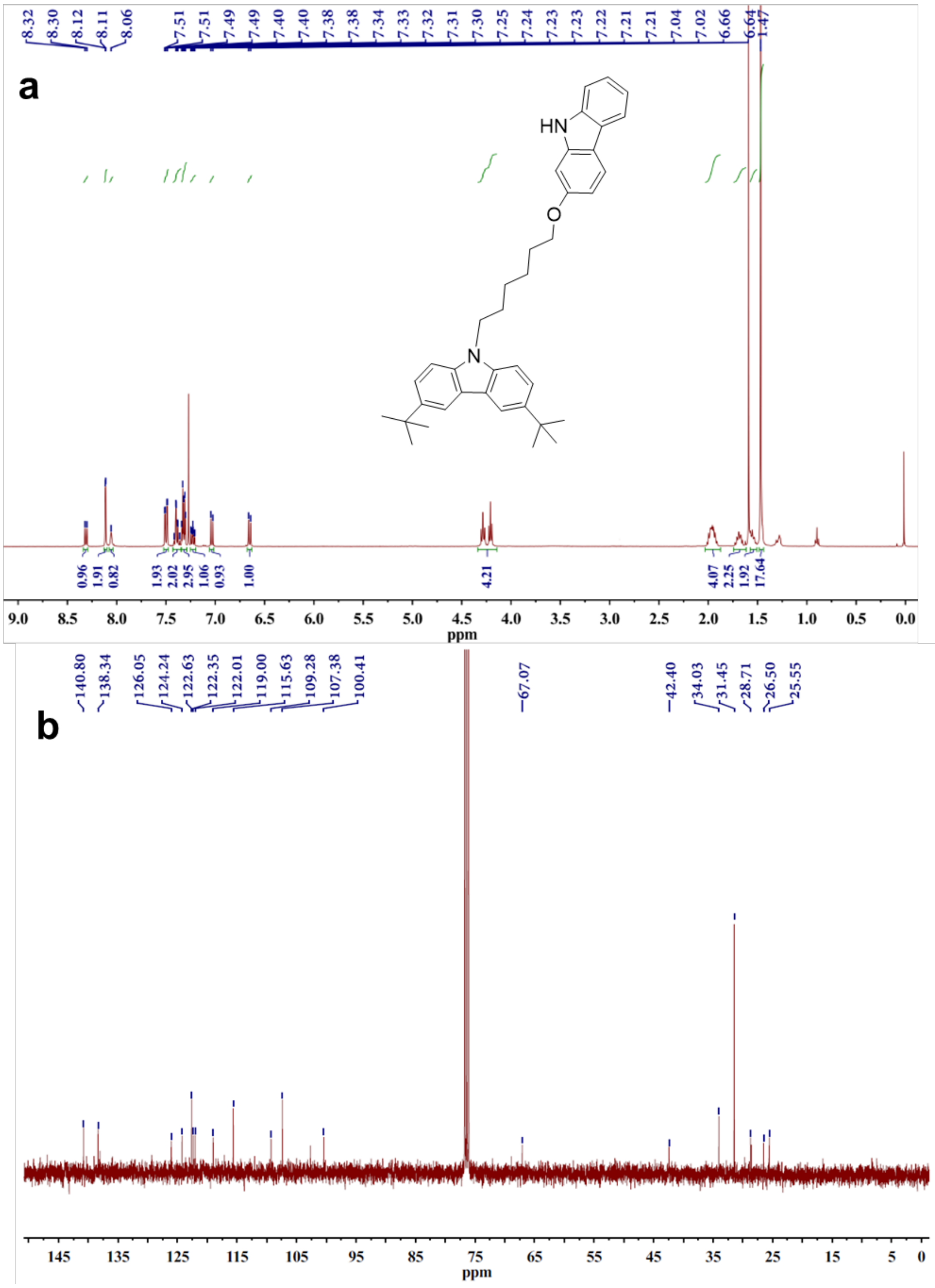

Figure S25. (a) ^{1}H NMR and (b) ^{13}C NMR spectra of 3.

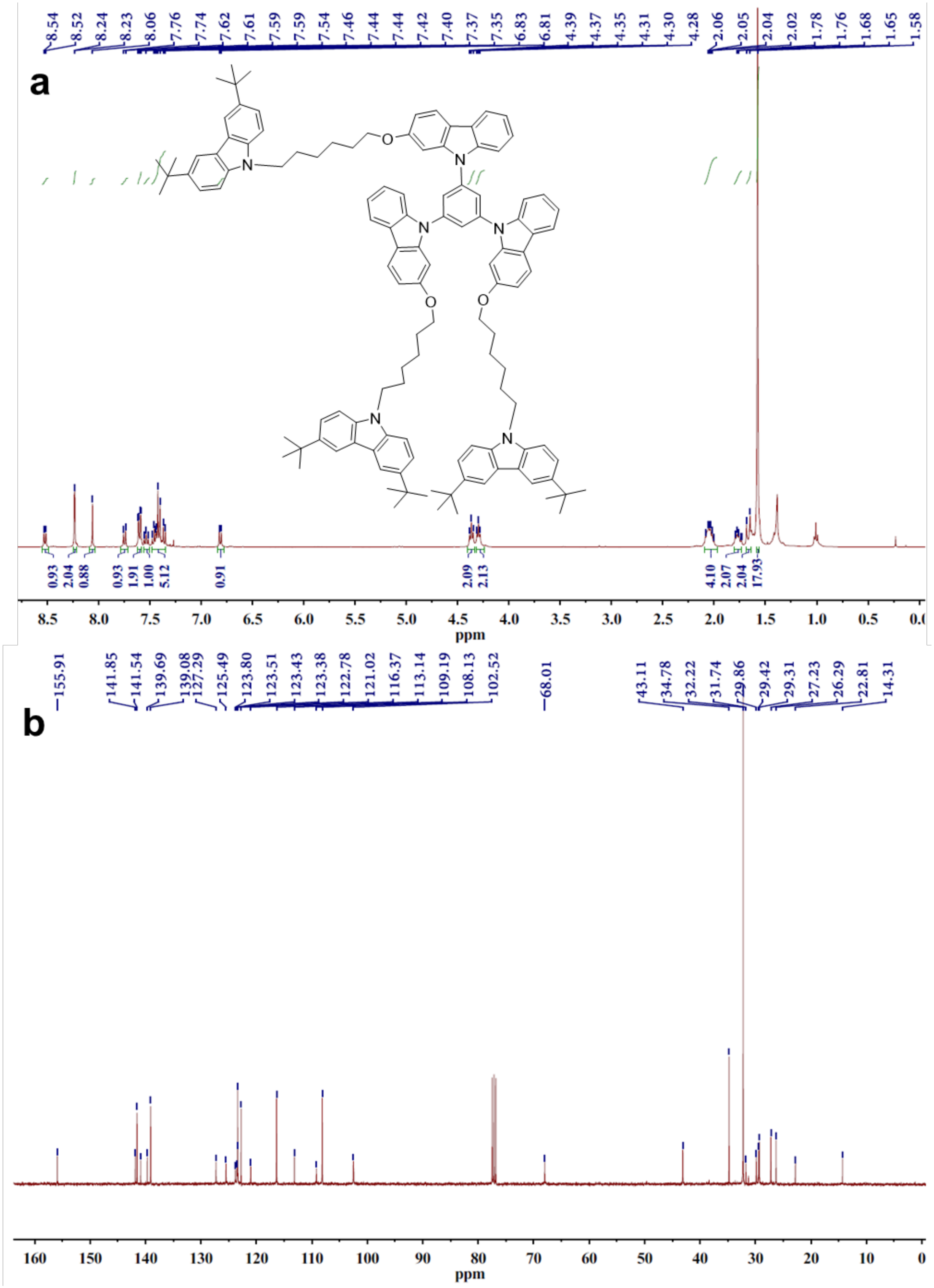

Figure S26. (a) ^{1}H NMR and (b) ^{13}C NMR spectra of 4.

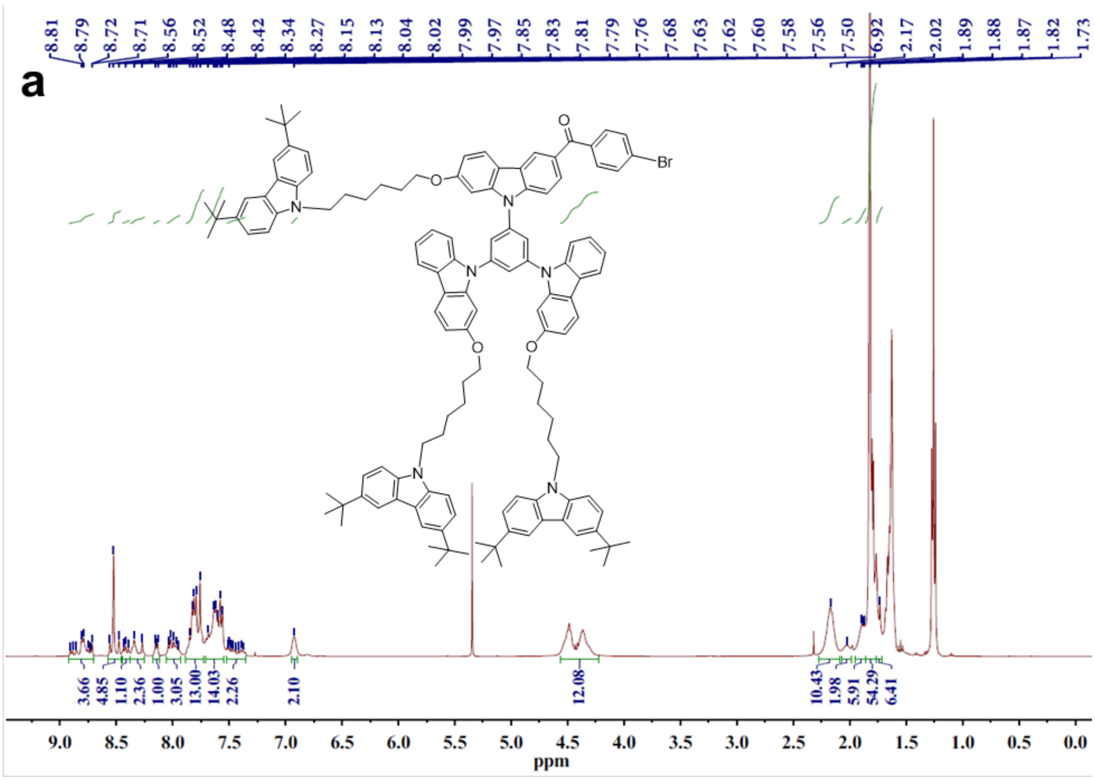

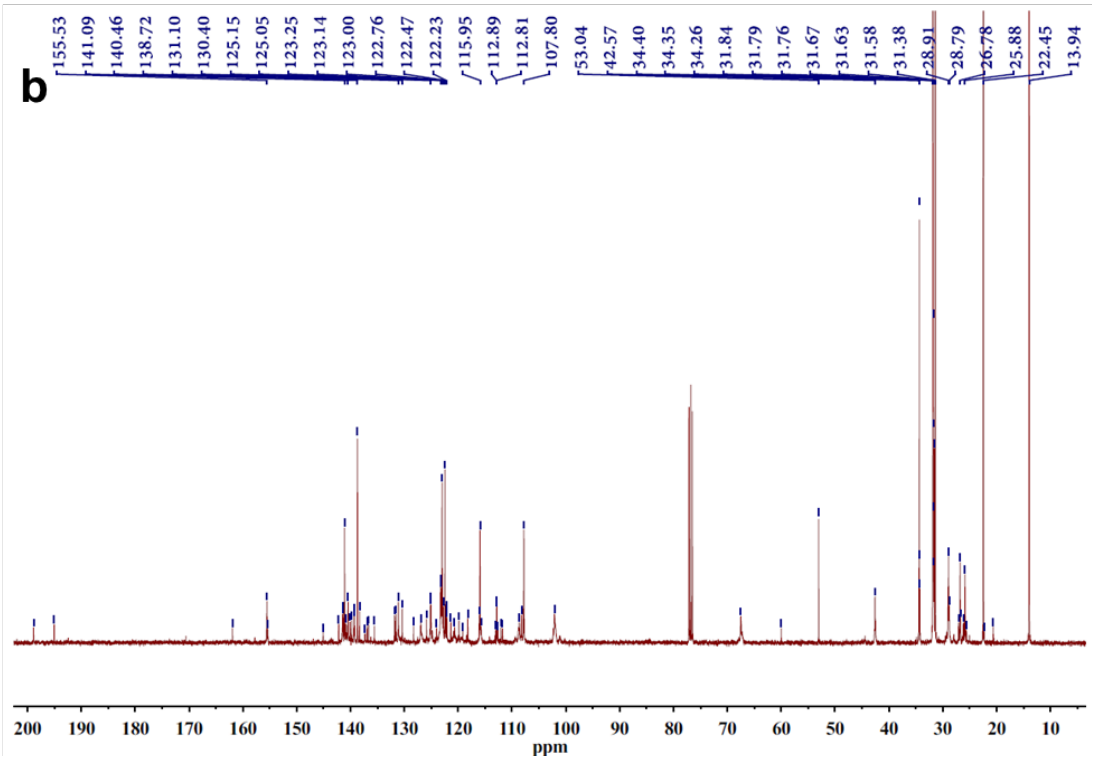

Figure S27. (a) ^{1}H NMR and (b) ^{13}C NMR spectra of 5.

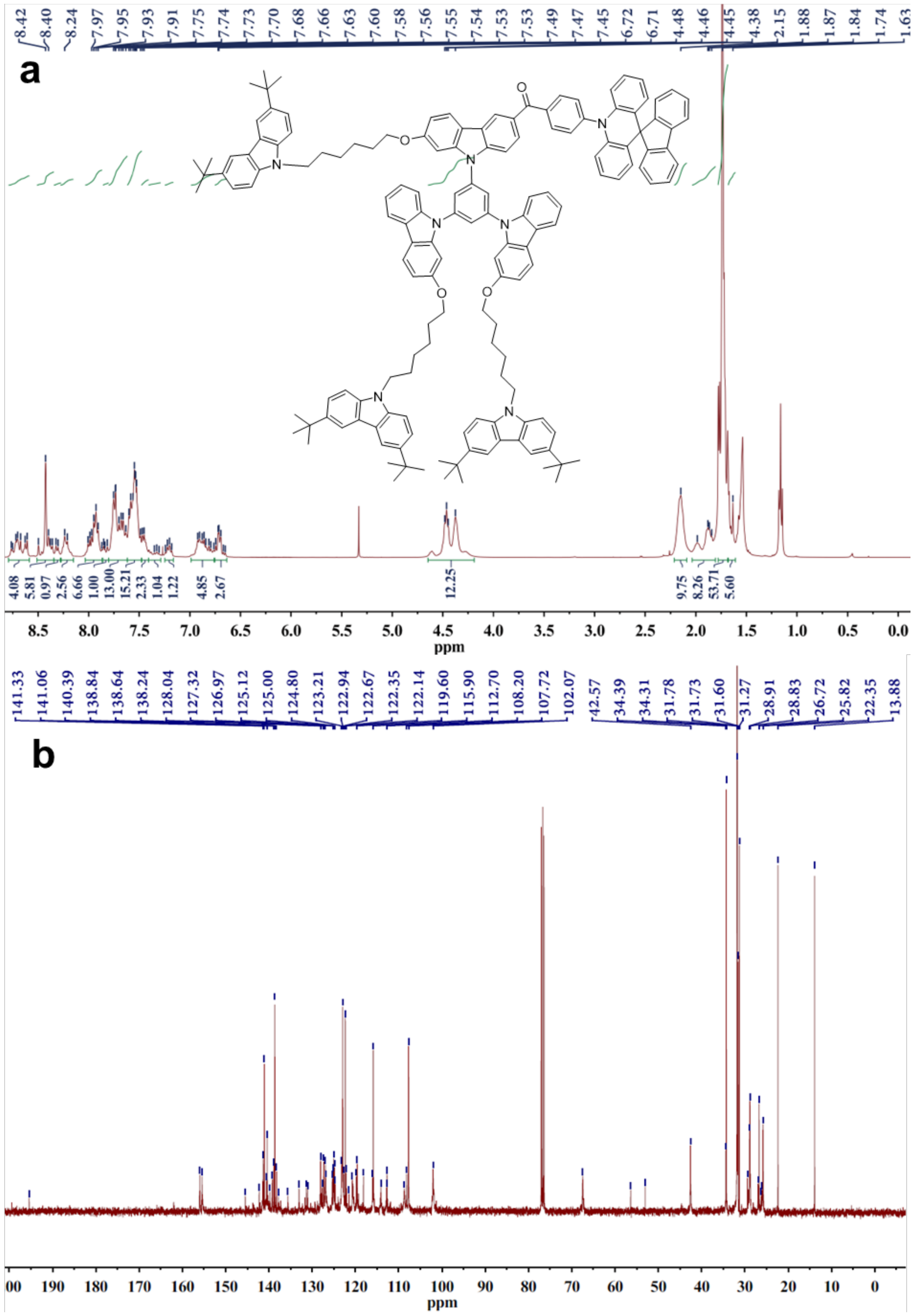

Figure S28. (a) ^{1}H NMR and (b) ^{13}C NMR spectra of dTC6-BPSAF.

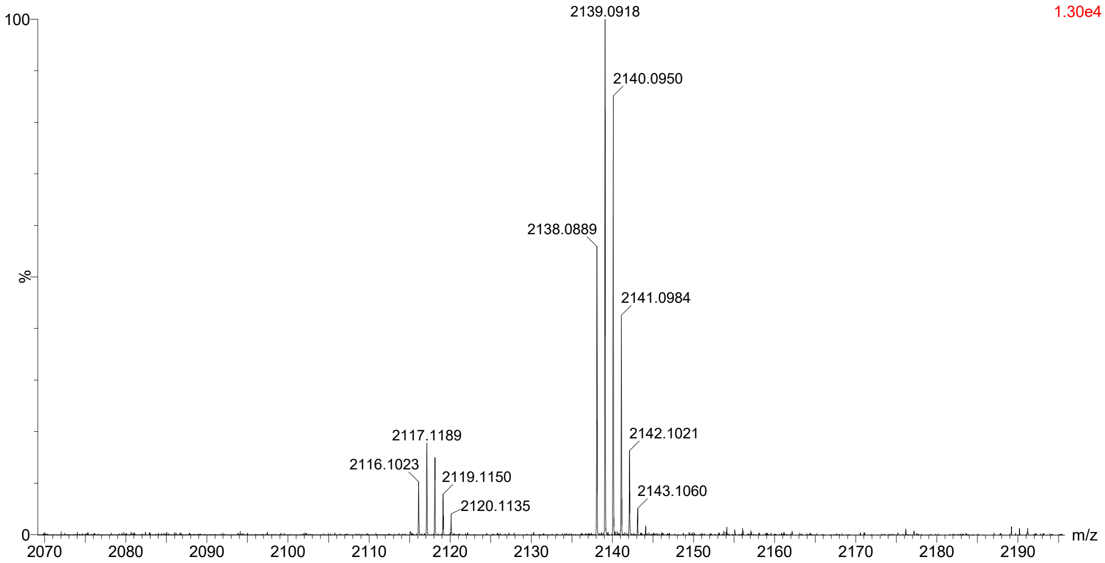

Figure S29. MALDI-TOF MS spectra of dTC6-BPSAF.

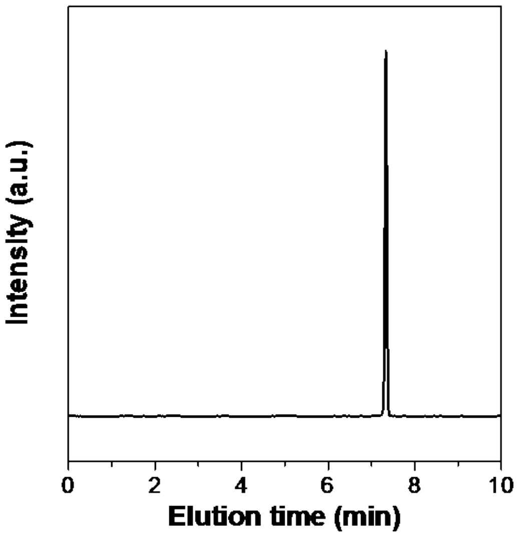

Figure S30. High-performance liquid chromatogram of the dTC6-BPSAF in acetonitrile/water mixtures.

References:

[1] S. E. Salih, J. K. Oleiwi, A. Mohammed.T, *Eng. Technol. J.* **2016**, *34*, 2838-2853.

[2] C. Pavon, M. Aldas, J. López-Martínez, S. Ferrándiz, *Polymers* **2020**, *12*, 334.

[3] J. C. K. d. Verney, M. F. S. Lima, D. M. Lenz, *Mater. Res.* **2008**, *11*, 447-451.
